# Evolution via recombination: Cell-to-cell contact facilitates larger recombination events in *Streptococcus pneumoniae*

**DOI:** 10.1371/journal.pgen.1007410

**Published:** 2018-06-13

**Authors:** Lauren A. Cowley, Fernanda C. Petersen, Roger Junges, Med Jimson D. Jimenez, Donald A. Morrison, William P. Hanage

**Affiliations:** 1 Department of Epidemiology, Harvard TH Chan School of Public Health, Boston, United States of America; 2 Department of Oral Biology, University of Oslo, Oslo, Norway; 3 Department of Biological Sciences, University of Illinois at Chicago, Chicago, United States of America; Université Paris Descartes, INSERM U1001, FRANCE

## Abstract

Homologous recombination in the genetic transformation model organism *Streptococcus pneumoniae* is thought to be important in the adaptation and evolution of this pathogen. While competent pneumococci are able to scavenge DNA added to laboratory cultures, large-scale transfers of multiple kb are rare under these conditions. We used whole genome sequencing (WGS) to map transfers in recombinants arising from contact of competent cells with non-competent ‘target’ cells, using strains with known genomes, distinguished by a total of ~16,000 SNPs. Experiments designed to explore the effect of environment on large scale recombination events used saturating purified donor DNA, short-term cell assemblages on Millipore filters, and mature biofilm mixed cultures. WGS of 22 recombinants for each environment mapped all SNPs that were identical between the recombinant and the donor but not the recipient. The mean recombination event size was found to be significantly larger in cell-to-cell contact cultures (4051 bp in filter assemblage and 3938 bp in biofilm co-culture *versus* 1815 bp with saturating DNA). Up to 5.8% of the genome was transferred, through 20 recombination events, to a single recipient, with the largest single event incorporating 29,971 bp. We also found that some recombination events are clustered, that these clusters are more likely to occur in cell-to-cell contact environments, and that they cause significantly increased linkage of genes as far apart as 60,000 bp. We conclude that pneumococcal evolution through homologous recombination is more likely to occur on a larger scale in environments that permit cell-to-cell contact.

## Author summary

Bacteria shuffle their genes far less often than humans do and genes or traits are more directly linked with the singular bacterial parent cell rather than the two parents that are involved in sexual reproduction. However, bacteria do occasionally have sex in the form of homologous recombination by taking up external DNA and incorporating it into their genomes. This happens far less regularly than sexual reproduction happens in human generations but is a known way that bacteria undergo ‘Horizontal gene transfer’. This means that genes can be acquired without being inherited. In this study we show that this form of horizontal gene transfer is more likely to happen in certain environments over others in *Streptococcus pneumoniae*. In particular, we show that this is more likely to happen in environments that closely mirror the nasopharynx which is the natural habitat of *S*. *pneumoniae*.

## Introduction

*Streptococcus pneumoniae* (the pneumococcus) is a paradigm for genetic transformation, in which cells become ‘competent’ for uptake of DNA through binding of a cognate competence stimulating peptide. The single stranded DNA that is taken up is then incorporated into the genome of the competent cell and can be detected as scattered segments of heterologous DNA. The consequences are of great relevance to human health, as they enable rapid adaptation to interventions such as antibiotic therapy and vaccination [1–4].

The pace of adaptation available through genetic transformation is limited both by the number of interactions and by the amount of DNA such an interaction can transfer, but neither of these parameters is well understood for any streptococcal species. Early estimates of the average size of recombined segments formed during transformation of pneumococcus *in vitro* varied over a range of 2–6 kb [5–7], although rare larger transfer events have since been detected *in vitro* with selective pressure [8]. WGS has enabled more comprehensive estimates of the sizes of DNA segments that can be transferred. In the first such global analysis in pneumococcus, determining the extent and frequency of replacements during transformation *in vitro* [9] with saturating concentrations of DNA, recombination events were limited to an average size of 2.3 kbp.

There is evidence however that recombination events observed in nature are larger. This could be a result of selection; for example, large-scale events were linked to vaccine escape variants with altered capsular loci, which emerged in the US from 2000–2007 [10]; one escape lineage (“P1”) displayed at least 9 replacements (sizes: 0.3, 0.5, 0.6,1.2, 2.3, 3.8, 26, 28, and 44 kb), all apparently from the same donor strain. A study of co-carriage of two strains in a single patient reported two large-scale multiple replacement events, each occurring within a 3-month time span [11]; one transferred three segments (sizes: 4.3, 13, 30 kb), while another transferred 6 segments (sizes: 0.8, 1.1, 4.3, 5.3, 6.5, and 20 kb). A survey of recombination events within a single geographically expansive clone of pneumococcus over 24 years mapped tracts of exchanged DNA over a wide range of sizes, from 2 to ~72,000 bp [1]. Similarly, two pneumococcal strains (PMEN3 and CGSP14) were identified that had acquired, respectively, 9 and 15 regions from PMEN1 or relatives, encompassing segments of 1.6–32 kbp or 4.6–22 kbp [4]. A survey of 173 isolates of a single lineage (CC320), mapped numerous recombination events, including an exchange of 78.8 kbp [12]. 228 ST127 strains from 3 lineages were reported to display 239 recombination events, with mean sizes varying widely among the lineages (4650, 21150, and 75156 bp) [13]. These examinations of products of gene transfer in nature are all retrospective analyses that are difficult to interpret clearly, as the strains were recovered after extended but poorly defined periods in which multiple exchange events might have accumulated. However, direct experimental studies of transformation in the natural environment are both rare and challenging to design.

**Fig 1:**
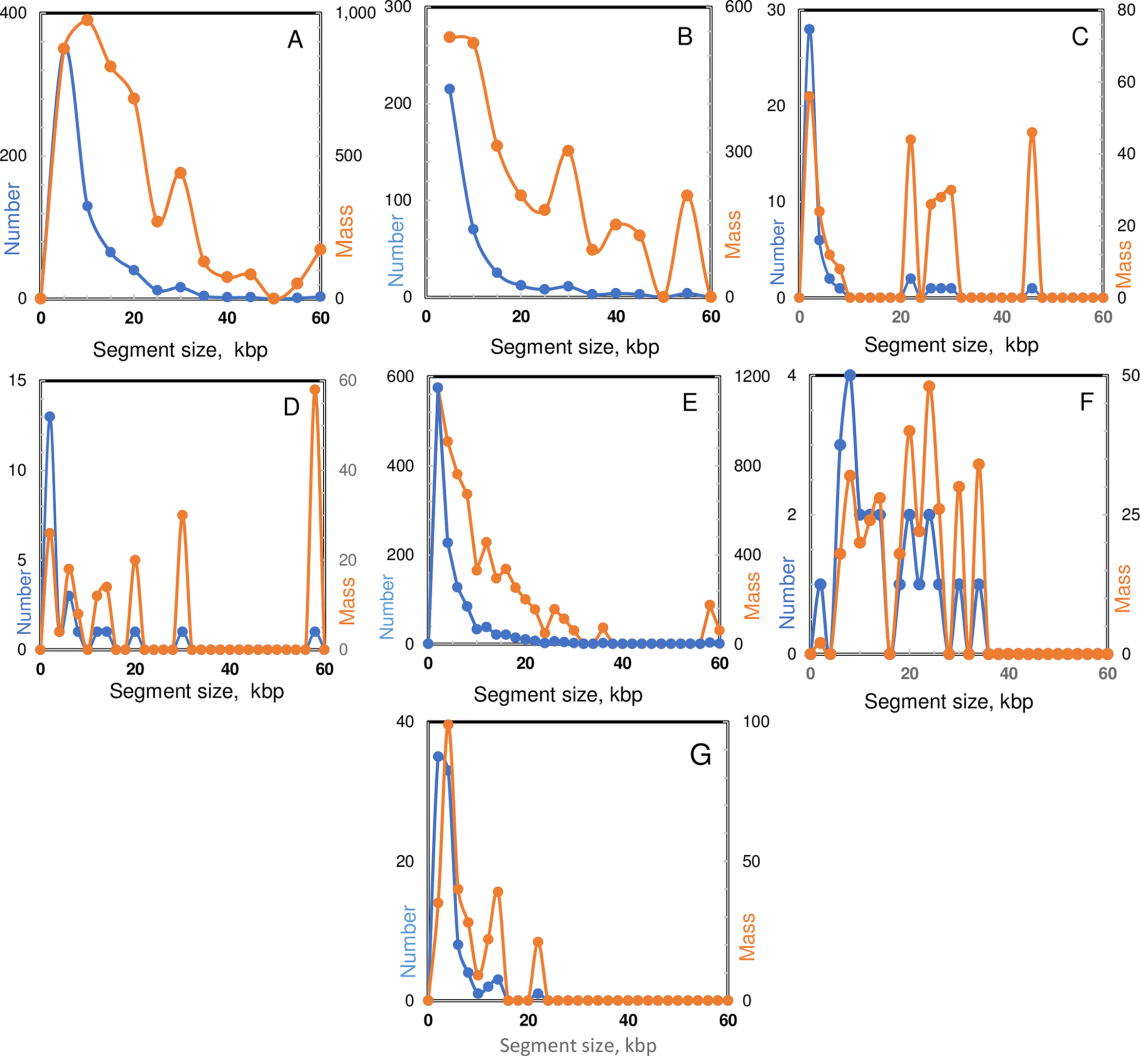
Recombination patterns reported for *S*. *pneumoniae* in nature and *in vitro*. Frequencies of recombination events as a function of segment size, plotted for six retrospective global analyses of exchange events in nature and one global analysis of products of genetic transformation *in vitro*. Recombinant segments were aggregated in 2-kbp increments. Panels display both the number of events in each size class (**blue**) and total mass of DNA (kbp) incorporated during those events (**orange**). Data extracted from cited publications. **A.** 173 strains of the PMEN4 lineage: 451 events total. [12]. **B.** 189 strains of the PMEN2 lineage: 616 events total [55]. **C.** Two vaccine escape strains: 27 events total [10]. **D.** Two ST13 strains from one patient: 23 events total [11]. **E.** 241 strains of the PMEN1 lineage: ~800 events total [56]. **F.** 426 strains receiving DNA from PMEN1 lineage: ~24 events total [4]. **G.** 84 strains of PMEN1 strain 23-F-R transformed by Kan^R^ DNA from strain TIGR4: 107 RSS events [9].

To compare reports of recombination in nature with what occurs *in vitro*, we collected length distributions documented in six studies of events in natural populations and in one study of events *in vitro* (Fig 1). Although there is variation in event-calling algorithms and biological histories of the strains examined, two common features of the natural population studies stand out. First, small recombination events are most common. Second, the bulk of the DNA transferred is carried in larger transferred segments. The proportion of DNA transferred in segments above 10 kb ranges between 70 and 90% in 5 of the 6 collected studies. In contrast, less than 12% of the DNA transferred *in vitro* is found in larger fragments.

Some of the larger recombination segments observed in nature transfer *cps* cassettes, which are as large as 30 kbp, make one-step shifts in serotype, and are likely to be under strong selection. However, it is not known if strong selection is also the basis for other large transfers persisting in wild lineages. While transfer of a *cps* locus *in vitro* is certainly possible, as demonstrated previously [8] for segments as large as 47.8 kbp, the low yield of such replacements (1 per 1000 closely linked single-gene transfers) leads us to expect that they should be more rare relative to small events than we observe in nature.

An increase in WGS of the pneumococcus has made it possible to map many recombination events occurring during the evolution of a lineage; the larger of these inferred events has often attracted comments on lengths that seem long for products of genetic transformation. The largest events have led to speculation about unknown transfer mechanisms or special circumstances of transformation not reproduced in the classical laboratory genetic transformation experiments. Two classes of explanation have been advanced to account for the contrast between the prevalence of large transfer segments in nature and their paucity *in vitro*. One attributes it to the selective filtering recombination, in which events must survive in order to be fixed in a lineage. The other proposes that there are unidentified special conditions of transformation in nature that generate an enrichment in large transfer segments, prior to selective filtering. Distinguishing between these explanations would require characterization of gene transfer products immediately after transfer in nature, or in suitably natural conditions, before intervention of selective filtering.

Bacterial physiology variation in nature, such as reduction in mismatch repair efficiency or altered levels of recombination proteins, could account for the observed difference. However, the recent recognition that gene transfer is especially efficient within biofilms [14] led us to speculate that the key distinction could be that in natural populations gene transfer often occurs in biofilms. A notable difference between the classical transformation of pneumococcus with purified DNA and the natural environment of the nasopharynx is the potential in the latter for direct cell-cell contact. The ability of competent pneumococcal cells to kill non-competent pneumococci on contact, through action of a competence-specific surface-bound lysin, CbpD, and other lysins [15] might create an enriched micro-environment affording a source of closely related, concentrated, and largely intact DNA. Our hypothesis is that transfers during *S*. *pneumoniae* cell-to-cell encounters are often qualitatively different from the cell-DNA encounters previously studied in the laboratory. This might yield larger recombination events, and that they more closely resemble those occurring during the habitual growth of pneumococci on host tissue surfaces in multicellular aggregates (biofilms) where interacting cells are anchored in close proximity. If correct, this hypothesis would offer explanations for both perplexing features of natural pneumococcal gene exchange–the increased size of individual recombination events and the simultaneous modifications at multiple genomic sites.

To examine the mechanism of pneumococcal gene transfer in settings that can allow experimental manipulation but better model the natural biofilm growth habitat, we designed pairs of densely labeled recipient/donor strains with genetic markers that enable precise monitoring of cell-cell interactions, detection of gene transfer events, and recovery of recombinant progeny for comprehensive mapping of the products of those events. We report here that global mapping of exchanges in recombinants recovered from two models of cell-cell interaction indicates significantly increased rates of co-transfer over large genetic distances during such encounters.

## Results

To trace recombination events with high resolution, a pair of well-marked pneumococcal strains was designed. Features comprised (1) >10,000 SNPs distinguishing the strains, (2) a donor that is incapable of developing competence, (3) a recipient whose competence development depends on exogenous competence pheromone peptide (CSP), and (4) markers to allow selection of recombinants. The recipient CP2204 was a *comA* derivative of the reference strain R6 that is incapable of secreting CSP and therefore depends on exogenous pheromone for competence development. It also carries the robust selective marker, Rif^R^. The donor was a competence defective *comE* deletion mutant that cannot sense CSP, with two selective markers, Spc^R^ and Nov^R^, introduced by transformation from R6 derivatives (S1 Table). The Spc^R^ and Nov^R^ markers are unlinked during transfer by transformation, as they are separated by ~800 kbp. The construction of this strain pair is summarized in Materials and Methods. After this pair proved incompatible in biofilm culture, a second competent (but *comA*^+^) recipient strain, R36AKan (a low-passage ancestor of R6) was substituted for use in mixed biofilm cultures, where competence would depend on endogenous elaboration of CSP by the recipient. Resequencing of the new derivatives showed that both recipients were distinguished from the donor by ~16,000 SNPs (15,954 in the case of CP2204 and 16,065 in the case of R36AKan). In the fratricide model of transformation, the cell taking up the DNA is termed the ‘attacker’ as it has lysed the other, which is termed the ‘victim’. Here we use the more general terms ‘recipient’ and ‘donor,’ which are independent of the mode of interaction in which transfer occurs. However, the reader will appreciate the different cellular roles in the interaction contemplated and that the donation of DNA is not ‘voluntary’.

### Varying recombination rates under different transformation conditions

Transformation events in nature likely occur among mixed populations such as those growing on an epithelial surface, often forming a biofilm. It is not clear what to expect for recombination in a biofilm. Biofilms frequently contain large quantities of DNA, which might compete with any DNA released during fratricidal attacks [16], but if they also include nucleases this DNA may be highly fragmented. High rates of gene transfer have been reported within experimental pneumococcal biofilms [14], but the nature of the responsible recombination events was not investigated in detail.

**Fig 2:**
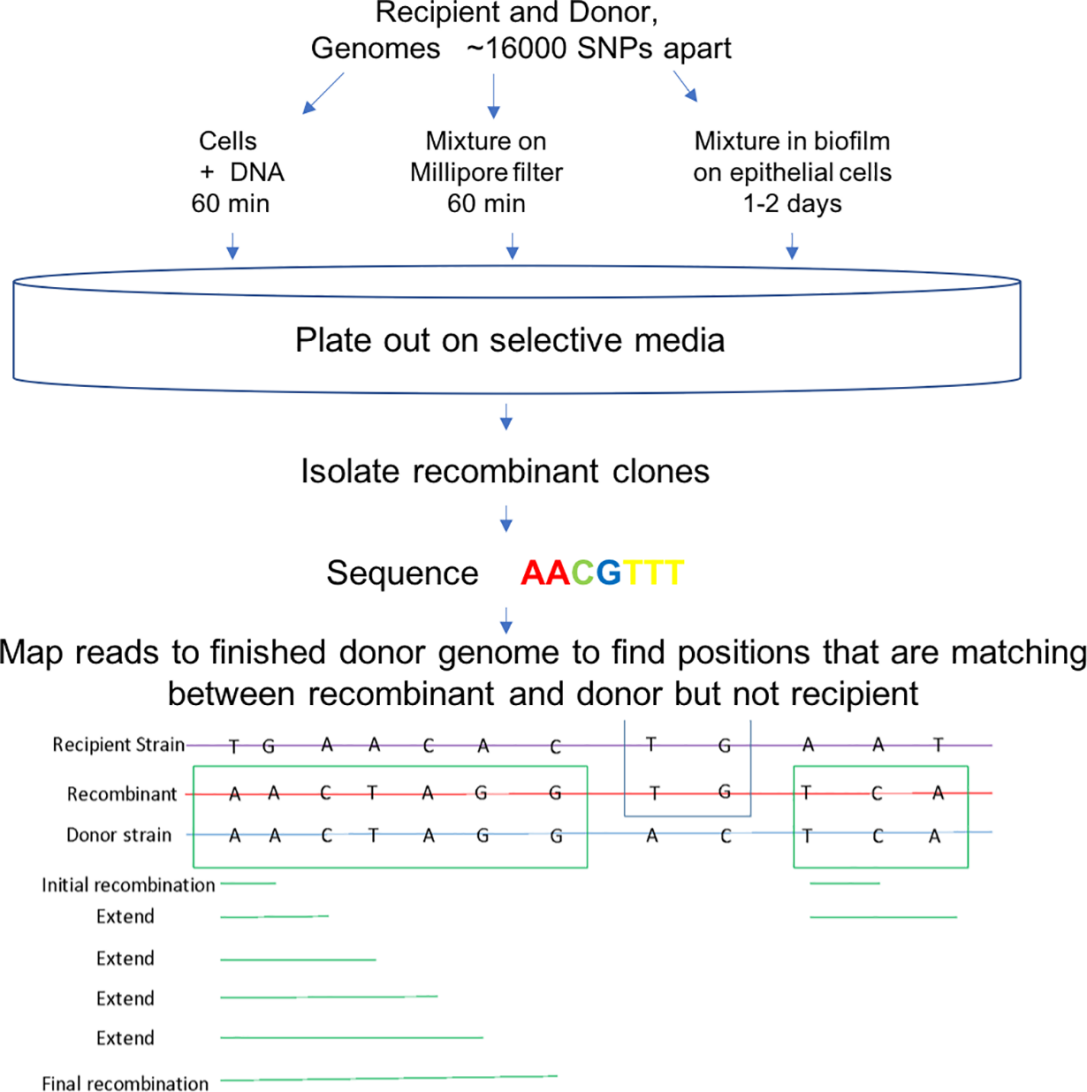
Schematic description of experimental workflow and data analysis representing the process of analysis for the entire project. Recipient and donor strains were known to be ~16000 SNPs apart and three experimental set-ups were conceived to test the effect of environment on recombination; saturating DNA, filter assemblage and biofilm co-culture. Recombinants were then recovered by antibiotic selection and sequenced. Final recombination events were determined by a mapping algorithm that identified variant regions (of the ~16000) which were matching between recombinant and donor but not recipient and therefore inferred to have been transferred.

Crosses were carried out under three different conditions: transformation with saturating purified DNA (similar to previous studies, [9]), transfer within mixed biofilm cultures (similar to Marks [14]), and transfer using a novel artificial biofilm-like assemblage (Fig 2). For direct comparison with DNA mediated transformation, a control experiment was carried out using the SNP-marked strain pair–CP2215 as source of 30-40-kb DNA fragments, and CP2204 as recipient. In transformation with saturating CP2215 DNA, copious Nov^R^ single-gene transformants were recovered, including detectable numbers of Nov^R^Spc^R^ double transformants (S2 Table). 22 independent double transformants were retained from the cross for mapping of transfer events. Although the transformation frequency for a single marker was only ~10^−4^, 0.5% of the Spc^R^ recombinants were also recombinant for the unlinked marker, Nov^R^. This high rate of co-transfer of two distant markers, termed congression [17], suggests that a minority of cells were highly competent, as this frequency would be expected to be no more than 0.01% for a recipient population of uniformly competent cells.

To ask whether large-scale recombination might be more common during cell-cell contact, we developed a synthetic biofilm-like assemblage in which competence could be controlled and recombinants directly and rapidly recovered. The assemblage consisted of culture mixtures collected from log-phase cultures onto a Millipore filter to a depth of ~20-cell thicknesses and incubated on the surface of an agar-solidified chemically defined medium (CDM). In this “filter assemblage”, recombinants were recovered after only an hour’s exposure to CSP (S3 Table). Among 2 x 10^10^ viable recipient cells recovered from the assemblage were 240,000 Spc^R^ transformants (a frequency of 1.2 x 10^−5^), among which 1% were also Nov^R^. The high congression rate again suggests that a minority of cells were highly competent. 22 independent Nov^R^Spc^R^ double transformants were retained from the cross for mapping of transfer events.

To ask whether large-scale recombination events also arise in biofilms, we recovered recombinants arising in the environment described previously [14], which uses co-culture in CDM to establish biofilms on a substrate of fixed confluent cells of a human lung epithelial cell line. We used a pair of strains with the known SNP differences described above (CP2215 and R36AKan), as initial trials showed that formation of biofilms with this pair of strains regularly yielded recombinants. Mature biofilms were developed after 48 h following inoculation of wells with 10,000 cells of each strain; but the recovered viable cells were a mixture of R36AKan and rare recombinants, with few remaining donors, consistent with widespread fratricide of CP2215 by R36AKan (S4 Table). Recovery of recombinants from the biofilm wells was variable, ranging from none to 0.1% of recipients (S4 Table). Despite the low rate of transformation, a high rate of congression, near 1%, again suggests the presence of rare competent cells. 22 independent recombinants were recovered for mapping of recombination events at the genomic scale. The retained clones represent selection for the recipient marker Kan^R^ in combination with Nov^R^ (14), Spc^R^ (3), or Cm^R^ (3) markers individually, as well as two cases of Nov^R^Spc^R^ double transformation.

### Characteristics of recombination events occurring under different transformation conditions

**Fig 3:**
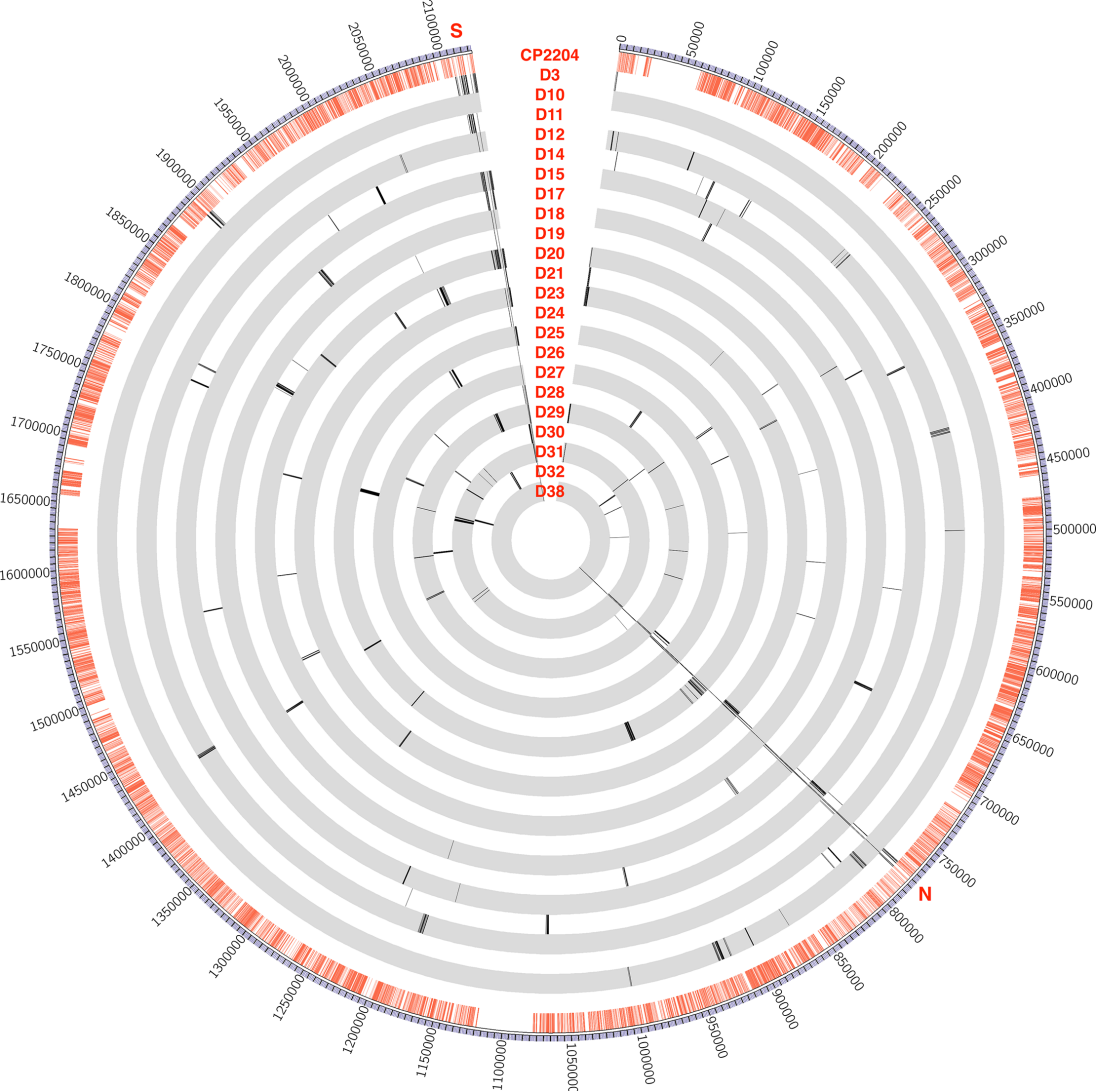
Recombination events in saturating DNA transformation. Circos plot visualizes the whole *S*. *pneumoniae* CP2215 genome with bp coordinates in 50-kb intervals labelled outside of the ring. Red lines in the first inner ring represent SNPs between strain CP2215 (donor strain) and CP2204 (recipient strain for saturating DNA experiments). Subsequent inner rings represent recombinants from the saturating DNA experiments. Lines on the inner rings represent SNPs transferred from donor to the recombinant. As expected, each recombinant strain carries one recombination event for each selected marker (Nov^R^ near 770 kbp and/or Spc^R^ near 212 kbp), plus some additional events nearby due to linkage, plus some events much farther away due to congression. Positions marked as S, N indicate positions of the Nov^R^ and Spc^R^ selective markers.

**Fig 4:**
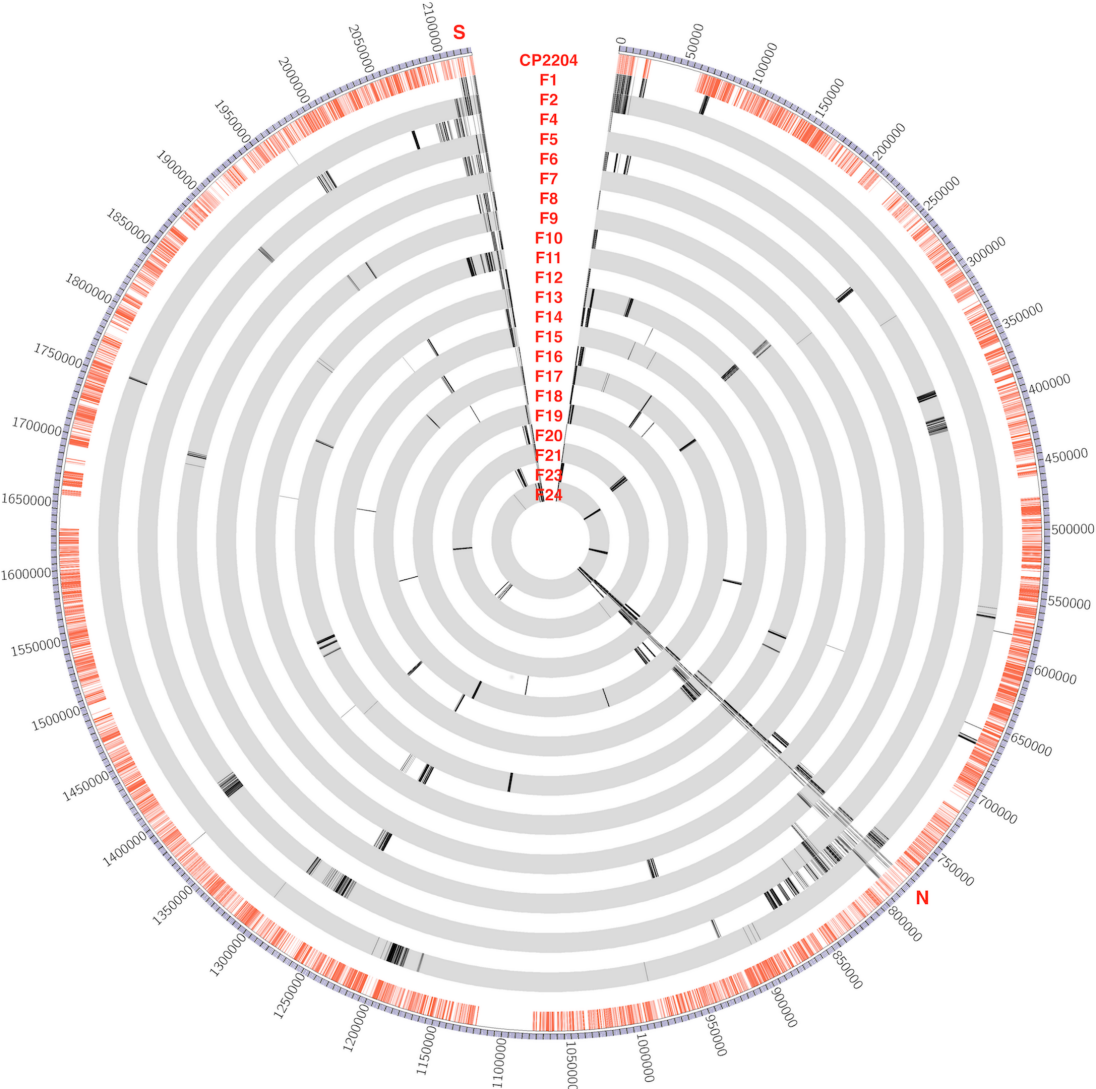
Recombination events in filter assemblage. Circos plot visualizes the whole *S*. *pneumoniae* CP2215 genome with bp coordinates in 50-kb intervals labelled outside of the ring. Red lines in the first inner ring represent SNPs between strain CP2215 (donor strain) and CP2204 (recipient strain for filter assemblage experiments). Subsequent inner rings represent recombinants from the filter assemblage experiments. Lines on the inner rings represent SNPs transferred from donor to the recombinant. As expected, each recombinant strain carries one recombination event for each selected marker (Nov^R^ near 770 kbp and/or Spc^R^ near 212 kbp), plus some additional events nearby due to linkage, plus some events much farther away due to congression. Positions marked as S, N indicate positions of the Nov^R^ and Spc^R^ selective markers.

**Fig 5:**
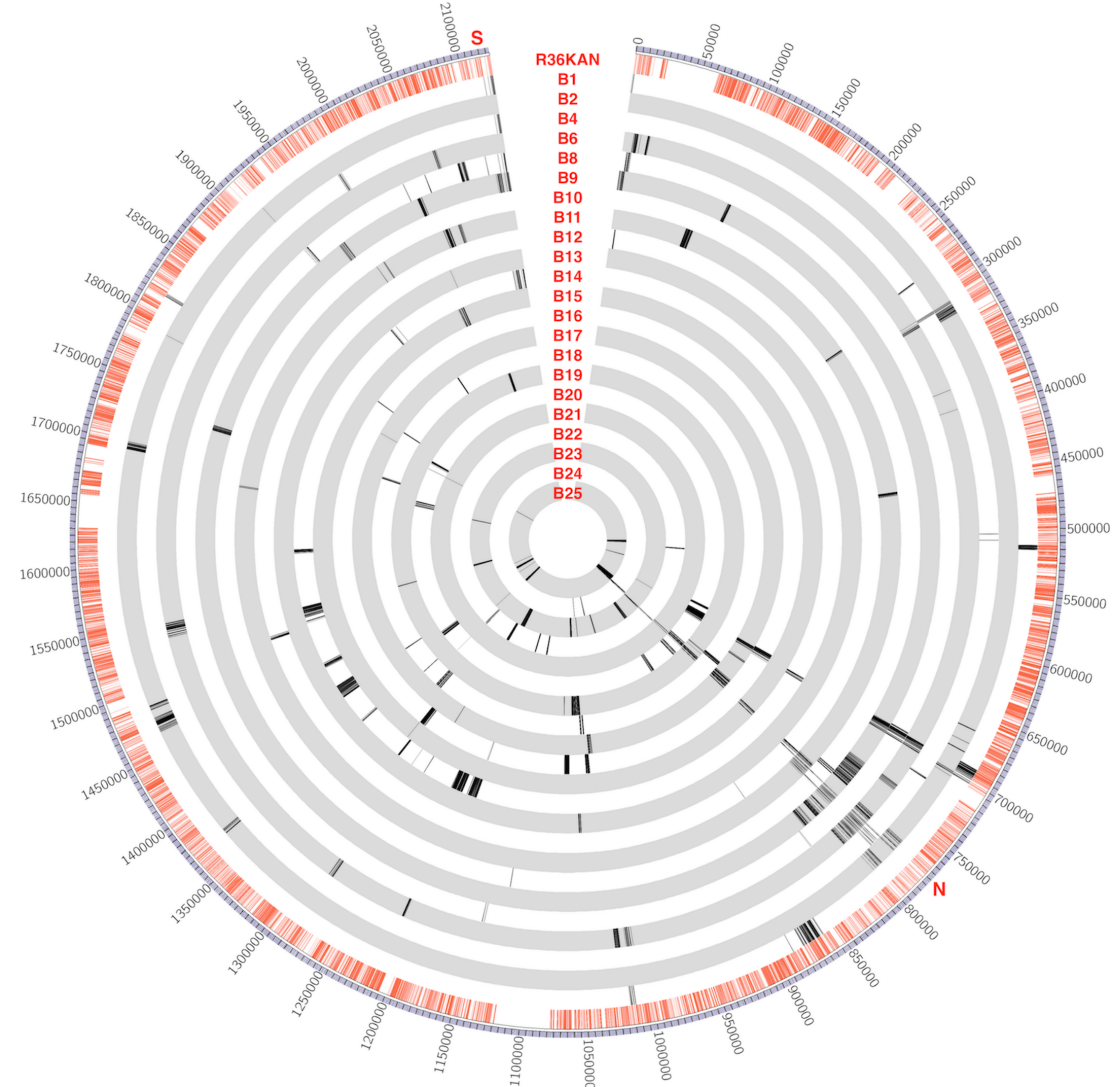
Recombination events in biofilm co-culture. Circos plot visualizes the whole *S*. *pneumoniae* CP2215 genome with bp coordinates in 50-kb intervals labelled outside of the ring. Red lines in the first inner ring represent SNPs between strain CP2215 (donor strain) and R36KAN (recipient strain for biofilm experiments). Subsequent inner rings represent recombinants from the biofilm experiments. Lines on the inner rings represent SNPs transferred from donor to the recombinant. As expected, each recombinant strain carries one recombination event for each selected marker (Nov^R^ near 770 kbp and/or Spc^R^ near 212 kbp), plus some additional events nearby due to linkage, plus some events much farther away due to congression. Positions marked as S, N indicate positions of the Nov^R^ and Spc^R^ selective markers.

Recombinants’ DNA was sequenced at 300X coverage and SNPs were identified by alignment to a finished genome of the donor strain (CP2215). Circos [18] plots of SNPs transferred from donor to recipient (Figs 3, 4 and 5) show that most of the SNPs transferred from donor to recipient occurred as clusters. The locations of the recombination events in each recombinant strain were identified by mapping contiguous sets of donor SNPs as described in the methods (Fig 2) and are listed in S5 Table. To compare the outcomes of recombination in the three types of cross, we assembled statistics on three readily quantified aspect of the results: the number of individual transfer events per recipient, the mean and standard deviation of sizes of individual transfer events, the number of events >10,000 bp, the total amount of DNA transferred to a recipient, and the percentage of the total differentiating donor/recipient SNPs that were transferred from the donor into the recombinant (S6 Table). These are minimum estimates, because in those genetic regions where the donor and recipient are identical, no recombination can be detected. There was substantial variation in the total amount of recombinant sequence acquired within all three types of experiments, with over 5% of the genome transferred in some recombinants, but only 0.04% in others. The number of events per recombinant also varied considerably, ranging from a low of 2 to a maximum of 20.

The recombination segment lengths were exponentially distributed in all three environments (S1 Fig); the corresponding per base probabilities of recombination are listed in Table 1. The majority of events were small, with rarer large events, consistent with previous work on transformation in nature and *in vitro* [9]. However, the mean sizes of events in the filter assemblage (4051 bp) and biofilm co-culture (3938 bp) crosses were larger than those in crosses using saturating DNA (1815 bp). Standard pairwise T-tests of the normally distributed mean recombination event size in the three equally sampled datasets showed that the filter assemblage and biofilm samples were not significantly different from each other (p = 0.7926), but the mean recombination event size of the saturating DNA experiments was significantly different to both the filter assemblage (p = 5.602 x 10^−9^) and biofilm (p = 3.885 x 10^−8^) cases.

**Table 1:** Summary of recombination events recovered from 66 recombinants in three experimental environments^a^.

**Environment**	**Total** **Events**	**Avg Number of** **Exchange** **Events /** **strain**	**Std dev**	**Mean recombination event size** **(95% CI)**	**Std** **Dev**	**Fixed per base probability** **(95% CI)**
Saturating DNA	186	8.45	4.48	1815 bp (802.51–2827.49)	2423	5.51 (3.54–12.46) ×10^−4^ bp^−1^
Biofilm	224	10.18	5.17	3938 bp (1986.15–5889.85)	4671	2.54 (1.70–5.00) ×10^−4^ bp^−1^
Filter Assemblage	255	11.59	5.04	4051 bp (2091.21–6010.79)	4690	2.47 (1.66–4.78) ×10^−4^ bp^−1^
**Environment**	**Events** **>10 kbp** **Average per strain**	**Std** **dev**	**Total transferred** **DNA, % genome per strain**	**Std dev**	**% SNPs** **Transferred,** **average**	**Std dev**
Saturating DNA	0.14	0.35	0.72%	0.34%	0.60%	0.43%
Biofilm	1.09	1.31	1.89%	1.54%	1.90%	1.57%
Filter Assemblage	1.45	1.18	2.21%	1.18%	1.55%	0.93%

a. Data from S6 Table.

While the average number of recombination events per cell increased only modestly under conditions for cell-cell interaction, the mean length of events doubled, and the total bp transferred per recombinant approximately tripled (Table 1). A clue to the source of this difference in total DNA transfer is the 8-10-fold increase in segments longer than 10 kbp.

**Fig 6:**
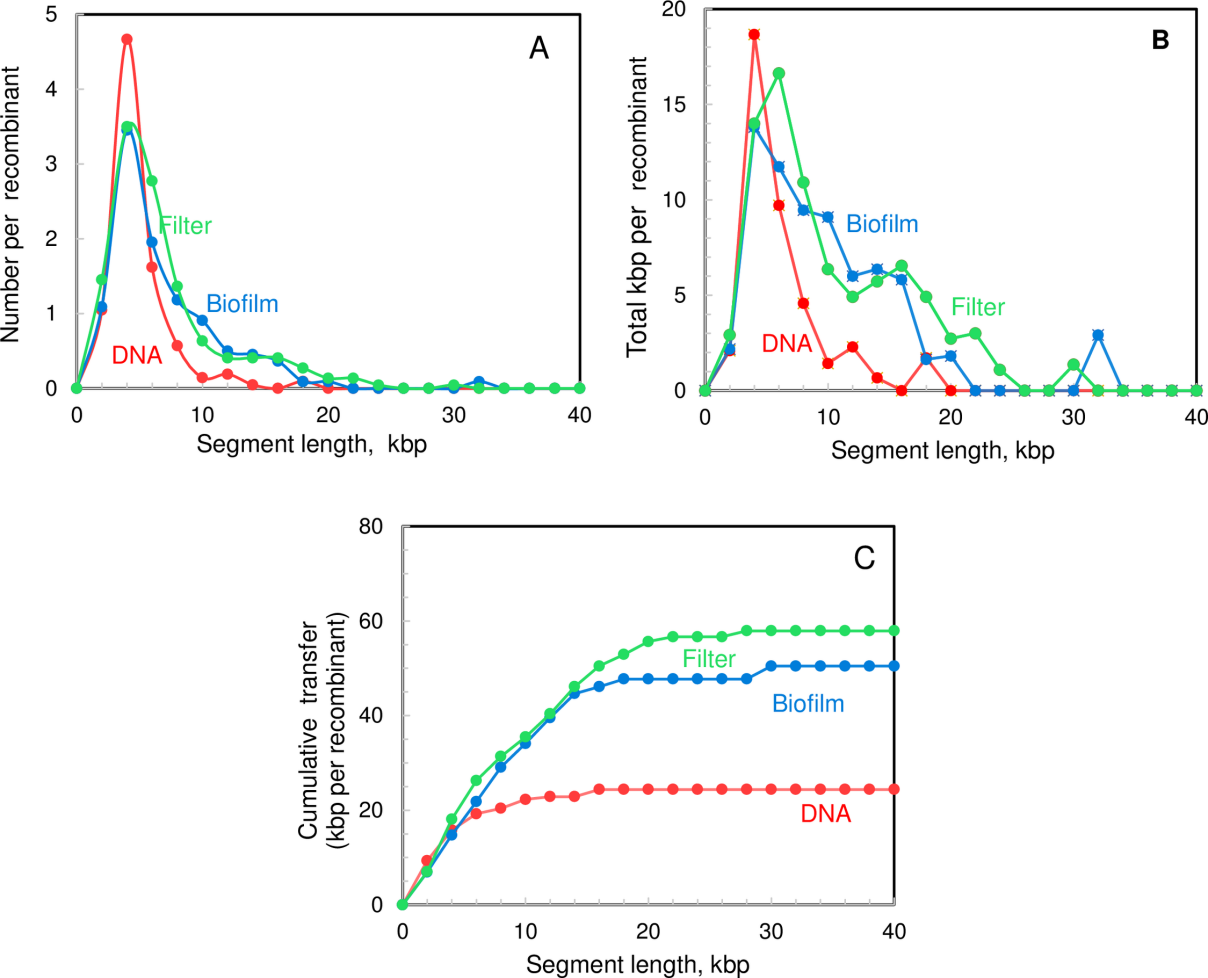
Distribution of recombination events during gene exchange *in vitro*, mediated by purified DNA or by cell-cell interactions. Recombination events mapped throughout the genomes of 22 recombinants from each cross format were sorted by length of recombinant segment (in 2-kbp increments) and aggregated to display average values per strain. **A.** Number of recombination events in each size class. **B**. Total amount of DNA transferred due to events in each size class. **C.** Cumulative amount of DNA incorporated due to events in all size classes up to the indicated segment length. **DNA**, transformation after treatment with CSP in CDM (CP2204 recipient, CP2215 donor); **Filter**, cell assemblage treated with CSP in CDM (CP2204 recipient, CP2215 donor); **Biofilm**, co-culture in CDM (R36A recipient, CP2215 donor).

To visualize the size dependence of these recombination events, the dependence of event number and transferred DNA content on segment size is shown in Fig 6. The frequency of smaller segments is the same for transfers in all three modes, but the increase in the amount of transferred DNA during cell-cell encounters occurred entirely through greatly (8-10-fold) increased numbers of larger (8–30 kbp) segments. We interpret this pattern as indicating that the cell-contact mode of genetic transformation increases the incorporation of long segments (and tracts of nearby segments) because the competent cells encounter longer DNA molecules, reducing premature interruption of processive transport by encounters with DNA ends.

Inspection of the recombination maps revealed exchange events near genome position 700,000 bp in 8 out of 22 recombinants recovered from biofilm co-culture, but in none of the recombinants from the other crosses. Notably, such events were not restricted to specific antibiotic selections, as they were observed in recombinants selected separately on novobiocin, chloramphenicol, and spectinomycin plates. These events were in the region of CtpA (a copper transporter P-type ATPase) and SpxB (pyruvate oxidase). It is possible that growth in biofilms may have imposed strong selective forces, such as oxidative stress and stationary phase cycles, which may have selected for recombinants in this region. Effects of sequence polymorphisms in SpxB have been investigated in relation to hydrogen peroxide production in *S*. *pneumoniae* [19]. However, effects of polymorphisms in CopA, SpxB, or both that could explain such a selection in biofilm cultures remain unknown.

### Linkage

**Fig 7:**
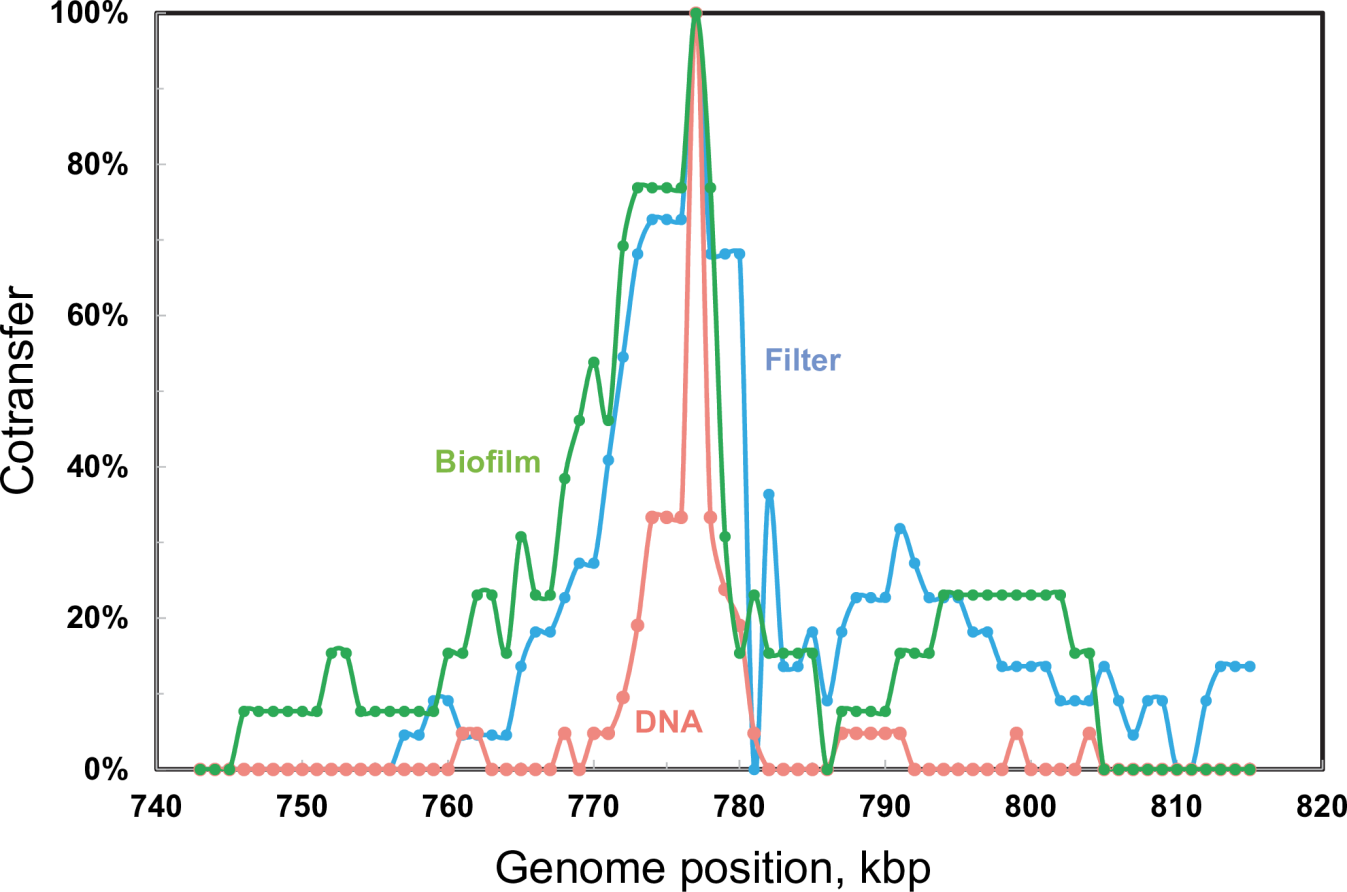
Expanded linkage during gene transfer in direct cell-cell interactions. Probability of inclusion in a recombinant segment is displayed as a function of genome position, within 40 kbp of the selected marker, *nov-1* (bp 777,151). Co-transfer rates for Nov^R^ transformants recovered from DNA transformation (**DNA**), short-term mixed strain assemblage (**Filter**), or biofilm co-culture (**Biofilm**), totalling 21, 22, and 14 strains, respectively. Background congression rates were 0.7%, 1.9%, and 2.2% respectively.

In crosses accomplished by genetic transformation, co-transfer of two distant markers into a single competent cell can occur independent of their proximity on the chromosome, by encounters with two separate DNA fragments, in the process termed congression. Linkage due to genetic proximity of two markers is revealed in practice by a frequency of co-transfer greater than the background rate of co-transfer by congression. In the present dataset, ~16,000 SNPs all served as genetic markers to evaluate the genomic extent of linkage to any selected marker. The incorporation of selective markers was typically accompanied by multiple additional transfers at widely separated sites throughout the genome, but tracts of tightly spaced recombination events were also observed (Figs 4 and 5). To visualize the effect of transfer mode on the span of linkage created by such tracts, Fig 7 displays the frequency with which each base in the vicinity of the *nov-1* marker was inherited by any of the Nov^R^ transformants in the DNA cross, the filter assemblage cross or the biofilm crosses. It is immediately apparent that the span of linkage to Nov^R^ is much greater in the cell-cell contact crosses than with saturating donor DNA.

### Comparison of the size distribution of recombination events to publicly available population samples

It has been suggested that differences between experimental recombination *in vitro* and what we see in nature may reflect the results of selection. For example, the major antigen in pneumococcus is determined by the capsule biosynthetic locus, which usually exceeds 10 kb in length. Large events at this locus are therefore more likely to change the serotype. In the PMEN1 dataset, containing over 240 isolates of a major intercontinentally disseminated resistant clone [1] the mean recombination event size was ~6kb. The largest event size detected in the PMEN1 dataset was 72,830 bp, while 20.3% of events were 10,000 bp or greater. We performed a similar analysis using the Gubbins algorithm [20] on 240 isolates collected by the CDC Active Bacterial Core (ABC) Surveillance system [21] and found a similar mean inferred recombination event size, 5589 bp (median event size 2963 bp, with a standard deviation of 7571). The largest event detected in the ABC dataset was 64,581 bp and 14.7% of events exceeded 10,000 bp.

To compare Gubbins-detected rates of recombination with our experiments, we must first investigate whether Gubbins can detect events as accurately as our mapping method. It should be noted that there are at least three systematic sources of bias toward detecting larger events in Gubbins [20]. First, as the algorithm detects elevated frequencies of SNPs in an isolate compared with an inferred ancestor, smaller regions would be less likely to be detected if donor/recipient SNPs are less dense in these samples than in our designed pairs of strains. This will raise the mean event size calculated both due to missing smaller events and due to omission of small intervening non-recombinant segments. Second, as discussed previously [20], the Gubbins algorithm is not able to differentiate clusters of recombination events that are separated by a few intervening recipient SNPs in the same way that our method does. Finally mismatch repair may have played a more important role in our study by preventing larger events. This could explain the clustering of events with intermediate recipient SNPs. Natural selection could take advantage of any larger recombination events arising from the failure of mismatch repair to provide a selective advantage that then survive in the population.

Comparison of Fig 1 with Figs 3, 4 and 5 shows that while cell-cell interaction increased mean segment size through greatly increased numbers of events in the 8–30 kbp range, it did not result in the even longer events reported elsewhere using Gubbins mapping tools. However, S5 Table details the results of applying the Gubbins algorithm to our dataset, with frequent clustering of individual events into very large events. The Gubbins algorithm repeatedly fails to recognize tracts of clustered individual events and instead groups them together, as seen, for example, in recombinant F4 where Gubbins groups 10 individual recombination events into one event over 90 kbp long. This provides one explanation as to why events are so long in retrospective evaluation of recombination events in published datasets: they may simply be groupings of smaller events that can be more accurately detected when the precise donor and recipient strains are known.

### Mechanism of transfers

When Mostowy et al. [22] examined recombination events in a single pneumococcal clone (PMEN1), they pointed out that three of the four known mechanisms of horizontal gene transfer were poor candidate explanations for the contrast between *in vitro* transformation and the apparent frequency of large recombination events in population samples. Transduction and transfer by gene transfer agent (GTA) particles [23] are strictly limited by the carrying capacity of the transfer particle, and, although integrated conjugative elements (ICEs) can transfer multiple linked genes, such transfer is usually restricted to a specific region of the chromosome. The remaining more likely responsible mechanism is natural genetic transformation, which is widespread in pneumococcus, transfers genes equally well from all parts of the genome, and uses a mechanism without inherent size limits or sequence specificity. It was suggested that large events could arise from a sporadic suppression of mismatch repair mechanisms. We now see that the cell-cell interaction previously missing in *in vitro* transformation experiments can itself explain most or all of the contrast.

The distribution of recombination events described here raises the question of what mechanism(s) might lead both to the widely-spaced events and to the closely clustered ones. The widely-scattered events must represent multiple parallel entry initiations, as cells remain competent for a limited time, and no single transport pore could import over half of a complete genome within this time. The process generating longer tracts of recombination events is less clear. They may be the result of the uptake of a single long strand of DNA, portions of which are then separated and independently inserted by homologous recombination. Alternatively, multiple pores may initiate parallel uptake events from multiple initiating scissions within a single 50-100kb domain of DNA. However, coordination to achieve the consistent strand choice would be difficult to explain.

A simple model that could account qualitatively for the major features of these patterns and is consistent with previously established properties of the transformation mechanism posits just 4 mechanistic steps:

1. Transport of DNA initiates at random sites within duplex DNA released upon fratricidal lysis of one or more marked non-competent cells, beginning by endonucleolytic scission and recruitment of the resulting 3’ end of one strand to an uptake channel, or ‘pore.’
2. The substrate strand is transported into the competent cell processively at ~80 bp/sec at each such uptake pore, accompanied by processive endonucleolytic digestion of its complement, leading to the uptake of single stranded DNA by the cell.
3. Import aborts with a low probability per base during processive transport, or on reaching the 5’ end of the substrate strand, but also upon inactivation of the pore during exit from competence.
4. Internalized single strands are subject to rare endonucleolytic scissions, yielding sub-fragments, which recombine independently into the resident chromosome(s), with a size-dependent efficiency.

Such a model would explain both the excess of small replacement events, due to nuclease activity within an unprotected cell lysate, and the existence of an upper limit to the sizes of the larger tracts of recombination events. Under this model, it is expected that different factors affect the yield of recombinant events in different ways, according to fragment size. At the low end, for example, it is known that the efficiency of recombination declines progressively for fragments below ~2 kb, but is constant above ~8 kb [24, 25]. Gaps in tracts of recombination segments would be a natural consequence of competition by daughter chromosome arms for segments of a single internalized donor strand. At the high end, in contrast, an upper limit on the size of recombination event tracts would be set by a finite rate of transport combined with a limited temporal competence ‘window’, a window that appears to be determined quite stringently both by retro-inhibition of the CSP response by the competence-specific protein DprA [26] and by lability of some competence effector proteins. Such a strand could approach 50–100 kb, if transport continued for 20 min at 80 bp/sec [27, 28].

## Discussion

These results show that large-scale gene transfer events do occur *in vitro* when the primary source of donor genes is living target cells, and supports the hypothesis that cell-to-cell contact facilitates larger recombination events.

Complex recombination events may be key to understanding the exceptionally large events reported for the PMEN1 and CC180 lineages [22]. Clusters of recombination events mapped near the *comE* and *nov-1* loci illustrate the potential for recombination episodes that simultaneously extensively modify the recipient genome over neighborhoods as large as 40–80 kb. Such recombination clusters appeared within recombinants from both filter assemblages and biofilm, but rarely or never during transformation with saturating DNA. Some recombination events identified within the PMEN1 and CC180 lineages were in the 20–80 kb range of sizes. Indeed, 50% of the total DNA transfer reported for each of these lineages was in events classified as 20 kb or larger. Many or all of these may represent clusters similar to those observed here but in which short internal recipient sequence blocks were obscured by the Gubbins detection algorithm in regions that are not densely populated enough with SNPs [20]. The same algorithm was used to re-analyze our experimental dataset to test event tract detection. The Gubbins software grouped many of these tracts into single large recombination events by ignoring the intervening recipient SNPs (S5 Table). This confirms that our mapping method provides a finer scale and more accurate measure of recombination events when the recipient and donor are known.

We note that the work here suggests that the frequency with which large recombination events occur, and hence the supply rate of selectable large-scale variation, may be higher than previously assumed within these antibiotic selected experiments for a limited number of samples (22 in each environment). Indeed, if these events are so relatively frequent that we readily observe them experimentally, why do they appear so comparatively infrequently in natural populations? One possible reason is that large insertions are more likely to have a negative fitness impact as a result of epistatic interactions with other parts of the genome [29]. In this scenario, large transfers happen at a high rate, but most such events are lost, leaving only the subset that provided some selective benefit or were neutral. There is previous evidence for this, in that in some cases making larger changes to the capsule locus appears to reduce growth rate [30], but not enough to indicate that it is a general mechanism. We note that taking up DNA from the environment may also serve purposes beyond adaptation, such as providing scarce nutrient [31] or helping with DNA repair [32]. If the majority of DNA is taken up for metabolism and rarely inserted into the genome, then competence would be maintained despite potential negative epistatic events of rare large recombination events. It is also important to remember that when cells come into contact with strains that are not as distantly related to themselves as the donor/recipient pair used here, recombination would introduce fewer SNPs and negative epistatic interactions would be less likely.

While this work suggests that larger fragments can be acquired under experimental conditions that more closely approximate natural co-colonization in the nasopharynx, it should be noted that the great majority of events remained relatively short, even in the biofilm. Importantly, this means that recombination is generally more likely to lead to the loss of accessory genes than their gain–consistent with a proposal that the function of homologous recombination is to remove parasitic elements [33]. However, our finding that larger recombination events occur alongside smaller ones suggests that while parasitic mobile elements can be effectively removed, this will happen at the same time as the acquisition of larger recombination events as part of the same process and from the same donor. If the majority of large recombination events have negative fitness consequences, the removal of the parasitic mobile element would be accompanied by the fitness cost of the large recombination event.

Does the pattern of large-scale recombination in population samples reflect the accumulation of separate transfer episodes, or coordinated simultaneous transfer of multiple DNA segments? Previous work has left this question open, as events had occurred within time windows of years [22] or months [10, 11]. This study shows that multiple and large transfers can occur within very much shorter temporal windows—a single 60-min competence cycle in the filter assemblages, and within 2 days in the experimental biofilm model. Thus, an inference that historical multiple large recombination events occurred within single or very few competence cycles is not unreasonable and explains instances where a single strain provided the source for multiple recombination events.

Within the wider context of other transformable bacteria, it is interesting to compare our results with those from similar genomic approaches to the study of recombination in Gram-negative pathogens. In particular, apparent clustering of insertion events into tracts has been reported in *Haemophilus influenzae* [34, 35] and *Helicobacter pylori* [36] (as well as previously in the pneumococcus [9]). However, the roles of biofilms have yet to be probed in experimental studies of these pathogens.

The present evidence that multiple recombination events can happen simultaneously means that we should not necessarily expect that recombination events are small, and occur at a rate of one per generation [37–40]. It is also clear that, although there was an obvious bias toward recombination events near the selected markers, simultaneous recombination events were widespread. This has resulted in a large proportion of the genome being transferred; indeed, in one isolate, as much as 5.8% (123,190 total bp) of the genome was transferred from donor to recipient, apparently in a single step, involving the insertion of 20 recombination segments. This amount is more than double the previous estimate of maximum recombination effect of recombination on the genome in *S*. *pneumoniae* (2.5% [9]); however a clinical long-term carriage study found that 7.8% of the genome could recombine [11]. The large amount of DNA that can transfer has obvious evolutionary implications and implies that evolutionary models for *S*. *pneumoniae* that attempt to encompass recombination should incorporate this scale of potential transformation.

The rate of recombination of a species is important for estimates of mutation and genomic change, and therefore the capability of a species to adapt to important selective pressures such as vaccines or antibiotics [41]. Current models of pneumococcal evolution incorporate regular short recombination events but not regular large recombination events [33, 42, 43]. While recent work on the impacts of recombination on epistasis indicated the proportion of the genome transferred in recombination as an important parameter [29], this work suggests it may be larger than would be assumed from previous experiments. There are several caveats of this observation that should be duly noted, measurements in nature will be effected by the loss of recombinants by negative selection, the potential enrichment of some recombinants by positive selection and a variable amount of growth of recipients that could also have biased these results. Nevertheless, this work suggests that the rate with which large recombination events occur is higher than has been suspected, and given that they appear to happen readily in experiment, but are comparatively rarely observed in nature [22], this may suggest that they are more likely to be selected against for reasons that will require further examination.

There are several limitations to this work. Our experimental model remains chemically and physically different from the natural environment of the nasopharynx, and we have worked with strains tractable to our purpose that may not be representative of the great diversity of pneumococcal lineages [44]. Indeed, our original pair did not effectively form a joint biofilm, and another suitable recipient strain had to be generated. This may suggest a reason for the observed and striking variation in recombination rate among pneumococcal lineages, where some seem to undergo no recombination at all despite possessing the necessary molecular machinery [45, 46]. This has been found to be associated with duration of carriage [13], but mixed biofilm formation is another variable that should be studied. Pneumococci that form mixed biofilms poorly with other strains may have fewer opportunities to acquire ‘foreign’ DNA in general and larger fragments in particular. Similarly if some lineages are more likely to co-colonize this may lead to a higher than expected rate of transfer between them [47].

Other limitations relate to aspects of the differing environments that vary in an unquantifiable way. For example, in the filter assemblage crosses, the ratio of donor cells to recipient cells was 1:5 (see S3 Table). Considering that the experiment was conducted for only 1h, the DNA ratio in the entire assemblage would remain mostly unchanged. In the biofilm context, this is harder to estimate, as the experiment lasted longer (48h). We can only take the input cell ratio as a guide to possibilities. The input ratio was 1:1 (see S4 Table). It has also occurred to us that the percentage of competent cells could influence the results; however, by our best estimates, competent cells were actually a minority in all three cross formats examined; so, if this aspect affects results, it may be expected to have the same effect in all three formats compared here.

The use of short read sequencing of the recombinants is a further limitation to the work. We have used long read sequencing and a finished genome of the donor strain to try to limit this but areas with too much genetic divergence (*e*.*g*., completely absent from the recipient genome) may encounter some mapping problems. Incomplete mapping can lead to misinterpretation of recombination event location or recombination start/stop position errors but cannot produce false positive recombination events.

### Conclusions

This study provides significant evidence that cell-to-cell contact, such as occurs naturally in a biofilm, significantly increases the likelihood of pneumococcal strains acquiring larger recombination events. We have also shown that these events are more likely to be clustered and characterized by short inter-recombination event regions in cell-to-cell contact environments. This has important implications for the study of pneumococcal evolution and may explain the apparent increased rate of recombination of some lineages of *S*. *pneumoniae* by an increased tendency to form biofilms. This work provides an explanation of why we observe larger recombination events when measured in the nasopharynx than what is measured in classical *in vitro* studies. In general, the broader implication of our study is that larger recombination events may depend on the opportunity for cell-cell contact, with minimal degradation of the acquired DNA.

## Materials and methods

### Bacterial strains and culture media

CP2204 is a CSP-dependent derivative of the R36A descendent CP2000 [48] (genotype: *malM bgl hex* Δ*cps rpsL1 hlpA*::GFP::CAT *comA*::*ermB rif*; phenotype: Hex^-^ Mal- Sm^R^ Cm^R^ Cps- GFP Em^R^ Rif^R^ ComA-). R36AKan is a derivative of the R36A ancestor of CP2000 that was marked by a Kan insertion in *rgg* (genotype: R36A but *rgg*::*kan*; phenotype: Hex^+^ Com^+^ Kan^R^). MD5037JANUS is a Δ*cps* derivative of the clinical isolate MD5037 [30]. The donor was CP2215, a non-transformable derivative of MD5037, which was chosen as being separated from R6 by >10,000 SNPs (genotype: MD5307 but Δ*cps*, *str-1 hlpA*::RFP::CAT *comE*::*spc nov-1;* phenotype: capsule-negative Sm^R^ Cm^R^ Spc^R^ Nov^R^ Com^-^). The sources and identities of selective markers used are listed in S1 Table. THY was Todd-Hewitt Broth (Becton Dickinson and Company, Le Pont de Claix, France) supplemented with 5 g/L yeast extract. CDM medium was as described [49], but supplemented before use with 1% choline and 1/100 volume of THY. Synthetic CSP1 [50] was obtained from Eurogentec at 95% purity and stored frozen in water at 100 μg/ml. Genomic donor DNA was purified from strain CP2215 as described previously [24]. For selection of recombinants, dilutions were incorporated in multi-layer THY agar plates, which allow expression of new genes before application of selective pressure [48]. Top agar contained selective compounds at selective levels of 100 μg/ml spectinomycin, 5 μg/ml rifampicin, 10 μg/ml chloramphenicol or novobiocin, or 800 μg/ml kanamycin.

### Sequencing of recombinants

Genomic DNA extracts were purified from log-phase THY cultures by bead beating according to the manfucturer’s protocol (Quick-DNA Fungal-Bacterial Microprep kit, Zymo). DNA concentration was measured using a Qubit fluorometer (Invitrogen, Grand Island, NY). Genomic DNA was normalized to 0.2ng per microliter prior to library preparation utilizing the Illumina Nextera XT kit. Briefly, 1 ng of the normalized DNA was subjected to enzymatic shearing and adapter ligation with the Illumina tagmentation enzyme. Adapted DNA was then amplified during which unique index sequences are added as well as the sequences required for cluster formation in sequencing. The resulting amplified library was cleaned by AMPure XP beads to remove short library fragments. Purified libraries were then checked with Agilent Tapestation and normalized for size and mass before pooling into a sequencing run. Libraries were sequenced on Illumina NextSeq with 2x150 bp sequencing reads generating approximately 7 million sequencing clusters and each recombinant sequenced to an average coverage of 300X.

### Nanopore sequencing and hybrid assembly of CP2215 (donor) genome

Nanopore sequencing was performed using the Ligation Sequencing Kit 1D. Briefly 1 μg of genomic DNA was sheared utilizing Covaris g-Tube to fragments of approximately 10 kb in length. The sheared DNA was end repaired and dA-Tailed according to protocol with NEBNext reagents. After AmpPure bead clean-up, adapters were ligated utilizing NEB Blunt T/A Ligase master mix. Following adapter ligation, the library is again bead cleaned and prepared for flow cell loading. Sequencing was performed for 48 hours on an R9.4 Spot ON flow cell. Total output for the Nanopore sequencing was > 40,000 passing reads. Illumina and Nanopore sequence data was combined for Hybrid Assemblies.

De novo assembly was performed using the Spades assembler [51] version 3.9 on both raw Illumina and Nanopore reads, with multiple k-mers specified as “-k 31,51,71,91”. Assembly of the strain resulted in one finished contig. Coverage levels were assessed by mapping raw Illumina reads back to the contig with BWA MEM [52] and computing the coverage as the number of reads aligning times the length of each read divided by the length of the contig. Automated annotations for the assembled contig were generated using Prokka [53].

### Recombination detection through SNP mapping

Fastq files of recombinants and recipients produced by WGS were quality filtered using trimmomatic [54]. The filtered fastq files (SRA BioProject:PRJNA448170) were mapped to the finished assembly of donor strain CP2215 (genbank Accession:CP028436) using SMALT. The consensus alignment was used to find SNPs between CP2215 and the two recipient strains (CP2204 and R36KAN). A customized python script was written to identify high quality SNPs (QUAL Filter > 50, Depth (DP) filter > 5, DP4 ratio filter > 0.75 (i.e. >75% of the forward/reverse reads need to be supporting the base call), Mapping Quality (MQ) filter > 30, Allele frequency (AF) filter < 0.05, Alternate allele frequency filter > 0.95, Strand bias > 0.001, Base Quality Bias > 0, Mapping Quality Bias >0, Tail distance bias > 0.001) in the alignment that were shared between donor and recombinant but not original recipient (full SNP lists available at https://github.com/laurencowley/Environmental-dependence-testing-on-recombination-in-Streptococcus-pneumoniae). This script then attempted to extend the match between donor and recombinant to adjacent SNP positions until the two sequences were no longer identical. Once the beginning and end (the last matching SNP position between the donor and recipient) of the recombination event had been established, the script extracted the sequence (Fig 2).

### Recombination detection through Gubbins for the ABC dataset

To examine Gubbins detection of rates of recombination within the ABC dataset, quality filtered fastq files for the 240 strains were mapped to the R6 reference genome to produce an alignment. This alignment was used in the recombination detection program Gubbins [20] which looks for elevated numbers of SNPs in an alignment to detect likely regions of recombination. Gubbins implements an approach to detect recombination that assumes a null evolutionary model on an alignment that has been base called to high quality SNP thresholds, with recombination events being identified as anomalous clusters of SNPs found close together. Hence in our analysis any regions with lower SNP quality could produce false positives that result in falsely recognized recombination regions. SNPs were called in this alignment using the same high quality thresholds as listed above. A custom python script extracted the recombination sizes from the Gubbins output to produce size distribution statistics.

### Recombination detection through Gubbins for our dataset

To examine Gubbins detection of rates of recombination for our dataset, the above described consensus alignment was used in the recombination detection program Gubbins [20], which looks for elevated numbers of SNPs in an alignment to detect likely regions of recombination. Gubbins results were parsed for concordance with our mapping method in identified recombination events and in their clustering.

## Supporting information

S1 Fig Distribution of recombination event sizes. (DOCX)

S2 Fig Cumulative total DNA transfer in the 3 environments. (DOCX)

S1 Table Source of genetic markers used. (DOCX)

S2 Table Transformation of recipient CP2204 with saturating genomic DNA. (DOCX)

S3 Table Gene transfer within filter assemblage. (DOCX)

S4 Table Recombination during co-culture in biofilm wells. (DOCX)

S5 Table Recombination events in 66 recombinant strains mapped by cross-match of reads to parental sequences. Cross formats: Dnn, saturating DNA; Fnn, filter assemblage; Bnn, biofilm.
(DOCX)

S6 Table Assembled statistics of the recombination events identified in each recombinant. (DOCX)
