# Supplementary material for: Evolution via recombination: Cell-to-cell contact facilitates larger recombination events in Streptococcus pneumoniae
Source: PLoS Genet. 2018 Jun 13;14(6):e1007410. doi: 10.1371/journal.pgen.1007410 (PMC6016952; doi:10.1371/journal.pgen.1007410)
Supplement: S1 Fig — (DOCX) [file pgen.1007410.s001.docx]

**Figure S1**. **Distribution of recombination event sizes** in base pairs (x axis) and count (y axis) in 22 recombinants from each environment. **A**. DNA transformation after treatment with CSP in CDM: CP2204 recipient, CP2215 donor. **B**. Filter assemblage treated with CSP in CDM: CP2204 recipient, CP2215 donor. **C**. Biofilm co-culture in CDM: R36AKAN recipient, CP2215 donor.
