# Supplementary material for: Evolution via recombination: Cell-to-cell contact facilitates larger recombination events in Streptococcus pneumoniae
Source: PLoS Genet. 2018 Jun 13;14(6):e1007410. doi: 10.1371/journal.pgen.1007410 (PMC6016952; doi:10.1371/journal.pgen.1007410)
Supplement: S2 Fig — (DOCX) [file pgen.1007410.s002.docx]

.

**Figure S2**. **Cumulative total DNA transfer in the 3 environments.** Cumulative total base-pair transfer (y axis) from donor to recipient genome, plotted by recombination event (x axis) for the Biofilm (red), filter assemblage (green) and Saturating DNA (blue) cross recombinants.
