# Supplementary material for: Evolution via recombination: Cell-to-cell contact facilitates larger recombination events in Streptococcus pneumoniae
Source: PLoS Genet. 2018 Jun 13;14(6):e1007410. doi: 10.1371/journal.pgen.1007410 (PMC6016952; doi:10.1371/journal.pgen.1007410)
Supplement: S1 Table — (DOCX) [file pgen.1007410.s003.docx]

**Table S1. Source of genetic markers used.**

|  | Location (bp) | |  |  |
| --- | --- | --- | --- | --- |
| Marker | In R6 | In CP2215 | Phenotype | Citation |
| *comE*::Spc | 2,033,994 | 2,117,236 | Spc^R^ | (Luo et al. 2003) |
| *comA*::ermB | 41,902 |  | Em^R^ | (Morrison et al. 1984) |
| *nov-1* (GyrBS127L) |  | 777,151 | Nov^R^ | (Cato and Guild.1968) |
| *hlpA*-GFP::CAT | 998,667 |  | Cm^R^ | (Kjos et al. 2015) |
| *hlpA*-Mkate::CAT | 998,667 | 1,038,281 | Cm^R^ | (Beilharz et al. 2015), |
| *rgg*::Kan | 941,641 |  | Kan^R^ | (Junges et al. 2017) |
| *rpoB* (Ser408Pro) | 1,751,671 |  | Rif^R^ | (H. Bai) |

Citations:

Beilharz K, van Raaphorst R, Kjos M, Veening JW. 2015. Red fluorescent proteins for gene expression and protein localization studies in *Streptococcus pneumoniae* and efficient transformation with DNA assembled via the Gibson Assembly Method. Applied and Environmental Microbiology. 81(20):7244-52.

Cato A, Guild WR. 1968. Transformation and DNA size: I. Activity of fragments of defined size and a fit to a random double cross-over model. Journal of Molecular Biology. 37(1):157-78.

Junges R, Salvadori G, Shekhar S, Åmdal HA, Periselneris JN, Chen T, Brown JS,

Petersen FC. 2017. A quorum-sensing system that regulates *Streptococcus pneumoniae*

biofilm formation and surface polysaccharide production. mSphere.

13;2(5).

Kjos M, Aprianto R, Fernandes VE, Andrew PW, van Strijp JA, Nijland R, Veening JW. 2015. Bright fluorescent *Streptococcus pneumoniae* for live-cell imaging of host-pathogen interactions. Journal of Bacteriology. 197(5):807-18.

Luo P, Li H, Morrison DA. 2003. ComX is a unique link between multiple quorum sensing outputs and competence in *Streptococcus pneumoniae*. Molecular Microbiology. 50(2):623-33.

Morrison DA, Trombe MC, Hayden MK, Waszak GA, Chen JD. 1984. Isolation of transformation-deficient *Streptococcus pneumoniae* mutants defective in control of competence, using insertion-duplication mutagenesis with the erythromycin resistance determinant of pAM beta 1. Journal of Bacteriology. 159(3):870-6.
