# Supplementary material for: Evolution via recombination: Cell-to-cell contact facilitates larger recombination events in Streptococcus pneumoniae
Source: PLoS Genet. 2018 Jun 13;14(6):e1007410. doi: 10.1371/journal.pgen.1007410 (PMC6016952; doi:10.1371/journal.pgen.1007410)
Supplement: S2 Table — (DOCX) [file pgen.1007410.s004.docx]

**Table S2. Transformation of recipient CP2204 with saturating genomic DNA.**

|  | gDNA |  | Recombinants ^a^ | | | Viable cells |
| --- | --- | --- | --- | --- | --- | --- |
| CSP | µg/ml | Initial OD | Nov^R^/µl | Spc^R^/µl | Nov^R^Spc^R^ /µl | Rif^R^/µl |
| Yes | 1 | 0.3 | 260 | 119 | 0.53^b^ | 1,600,000 |
| No | 1 | 0,3 | 0 | 0 | 0 | 500,000 |
|  |  |  |  |  |  |  |

(a) Culture CP2204 was grown in THY at 37° C to OD 0.2. After resuspending cells in cold CDM (acidified with 10mM HCl) at final OD_550_ values of 0.3, they were supplemented with CP2215 genomic DNA (1 ng/µl ), CSP as indicated (100 ng/mL), 0.5 mM CaCl_2_, and 0.02% BSA, then incubated 60 min at 37° C before dilution into THY containing DNase I, further incubation at 37° C for 90 min, and plating in THY agar with selection of Rif^R^ recombinants expressing Nov^R^ and/or Spc^R^.

(b) Twenty-two Rif^R^ Nov^R^Spc^R^ clones were picked and re-plated for single colonies; sub-clones were then stocked from each primary isolate. Rif^R^ Nov^R^ *comE*::Spc^R^ double recombinant stocks were named as Dnn.
