# Supplementary material for: Evolution via recombination: Cell-to-cell contact facilitates larger recombination events in Streptococcus pneumoniae
Source: PLoS Genet. 2018 Jun 13;14(6):e1007410. doi: 10.1371/journal.pgen.1007410 (PMC6016952; doi:10.1371/journal.pgen.1007410)
Supplement: S3 Table — (DOCX) [file pgen.1007410.s005.docx]

**Table S3**. **Gene transfer within filter assemblage.**

.

|  | |  | Recombinants ^a^ | | | Viable cells  / µl |
| --- | --- | --- | --- | --- | --- | --- |
| CSP | OD_550_ | | Nov^R^/ µl | Spc^R^/ µl | Nov^R^Spc^R^/ µl |  |
| Yes | | 0.79 | 63 | 24 | 0.252 ^b^ | 1,800,000 |
| No | | 0.82 | 0 | 0 | 0 | 4,000,000 |

1. Cultures of strains CP2204 and CP2215 were grown in THY at 37° C to OD 0.2, mixed at a 5:1 ratio, and passed through a Millipore HABG047 0.45-µfilter to concentrate the cells on the filter surface. After incubation of the filter inverted on the surface of CDM agar spread with 50 µl of a CSP stock (100 µg /mL) in a candle jar at 34° C for 60 min, cells were resuspended in 10 mL THY by use of a rubber disposable cell scraper. The OD_550_ was recorded before further dilution into THY containing DNase I, incubation at 37° C for 90 min, and plating in THY agar alone or with selection of Rif^R^ recombinants expressing Nov^R^ and/or Spc^R^.
2. Twenty-two Rif^R^ Nov^R^Spc^R^ clones were picked from selection plates and re-plated for single colonies; sub-clones were then stocked from each primary isolate. Rif^R^ Nov^R^ *comE*::Spc^R^ double recombinant stocks were named as Fnn.
