# Supplementary material for: Evolution via recombination: Cell-to-cell contact facilitates larger recombination events in Streptococcus pneumoniae
Source: PLoS Genet. 2018 Jun 13;14(6):e1007410. doi: 10.1371/journal.pgen.1007410 (PMC6016952; doi:10.1371/journal.pgen.1007410)
Supplement: S4 Table — (DOCX) [file pgen.1007410.s006.docx]

**Table S4. Recombination during co-culture in biofilm wells.**

| Well ^a^ | Harvest time, h^b^ | VC  /µl^c^ | Kan^R^Nov^R^  /ml | KN kept^d^ | Kan^R^Spc^R^  /ml | KS kept^d^ | Kan^R^Spc^R^Nov^R^  /ml | KNS kept^d^ | Kan^R^Cm^R^  /ml | KC kept^d^ |
| --- | --- | --- | --- | --- | --- | --- | --- | --- | --- | --- |
| P4 | 72 | 30000 | 28000 | **B17** | 9000 | **B1** |  |  | 50000 |  |
| P5 | 72 | 1000 | 4000 | **B2** | 0 |  | 0 |  | 3000 | **B18** |
| Q3 | 72 | 200 | 12000 |  | 5000 | **B14** | 30 | **B4** | 10000 | **B13** |
| Q5 | 72 | 200 | 8000 | **B15** | - |  | 30 | **B9** | 6000 |  |
| Q6 | 72 | - | 10000 | **B6** | 5000 | **B8** | 30 |  | 400 | **B16** |
| R4 | 24 | 60000 | 60000 | **B19** | - |  | - |  | - |  |
| R5 | 24 | 40000 | 8000 | **B10** | - |  | - |  | - |  |
| R6 | 24 | 30000 | 4000 | **B20** | - |  | - |  | - |  |
| S4 | 24 | 30000 | 36000 | **B11** | - |  | - |  | - |  |
| S5 | 24 | 40000 | 2500 | **B21** | - |  | - |  | - |  |
| S6 | 24 | 40000 | 4000 | **B12** | - |  | - |  | - |  |
| T3 | 48 | 2000 | 220 | **B22** | - |  | - |  | - |  |
| T4 | 48 | - | 40 | **B23** | - |  | - |  | - |  |
| U3 | 48 | 30000 | 40 | **B24** | - |  | - |  | - |  |
| U4 | 48 | 200000 | 20 | **B25** | - |  | - |  | - |  |

**(a)** To assemble biofilms, strains R36AKan (recipient) and CP 2215 (donor) without any exogenous CSP were mixed at 20,000 CFU/ml in CDM. 0.5-ml volumes were incubated at 34° C with CO_2_ in 24-well plates, with medium replaced every 12 h. Medium exchange used a large-bore 5-ml disposable pipette, removing ~90% of supernatant fluid in each medium change. Wells were prepared with a para-formaldehyde-fixed confluent A549 cell line substrate, according to Marks et al. (2011). -, not done.

**(b)** After 24, 48, or 72 h of continuous culture, adherent cells were scraped off the well bottom, suspended in 10% glycerol/THY, sonicated twice for 2 sec, and frozen/thawed (-80° C / 4° C) thrice. The thawed cells were vortexed thoroughly, and clarified by a 1-min 1,000xG spin. Finally, the supernatant cells were diluted and plated in selective THY agar.

**(c)** VC, total viable CFU.

**(d)** A single colony obtained from each well and selective medium was streaked on blood agar, and a sub-clone stored at -80°C for sequence analysis. Individual sub-clones from each selection that was subjected to sequence analysis were named Bnn as indicated in the ‘kept’ columns (K, Kan^R^; N, Nov^R^, C, Cm^R^).
