# Supplementary material for: Evolution via recombination: Cell-to-cell contact facilitates larger recombination events in Streptococcus pneumoniae
Source: PLoS Genet. 2018 Jun 13;14(6):e1007410. doi: 10.1371/journal.pgen.1007410 (PMC6016952; doi:10.1371/journal.pgen.1007410)
Supplement: S5 Table — Cross formats: Dnn, saturating DNA; Fnn, filter assemblage; Bnn, biofilm. (DOCX) [file pgen.1007410.s007.docx]

**Table S5. Recombination events in 66 recombinant strains mapped by cross-match of reads to parental sequences. Cross formats: Dnn, saturating DNA; Fnn, filter assemblage; Bnn, biofilm.** Positions of recombination segments determined as described in Fig. 2.

(a) Recombinant strain.

(b) **S**tart of recombination event in genome (bp).

(c) End of recombination event in genome (bp).

(d) Gubbins concordance. M = exact match in event prediction between mapping method (Fig. 2) and Gubbins; C = events clustered into a single event (box) by Gubbins; Blank = event not reported by Gubbins.

| **a** | **b** | **c** | **d** | **a** | **b** | **c** | **d** | **a** | **b** | **c** | **d** |
| --- | --- | --- | --- | --- | --- | --- | --- | --- | --- | --- | --- |
| D3 | 95 | 878 |  | F1 | 95 | 14149 |  | B1 | 95 | 878 |  |
| D3 | 772467 | 777940 |  | F1 | 20372 | 23674 | C | B1 | 512071 | 515615 | M |
| D3 | 1890651 | 1895945 | M | F1 | 69021 | 69215 | C | B1 | 689848 | 701685 |  |
| D3 | 2107330 | 2122425 |  | F1 | 69345 | 72612 | C | B1 | 860683 | 868356 |  |
| D10 | 777033 | 777150 | C | F1 | 579102 | 579345 | M | B1 | 868508 | 870946 |  |
| D10 | 791264 | 795395 | C | F1 | 654018 | 654225 | C | B1 | 871352 | 878140 |  |
| D10 | 868322 | 868323 |  | F1 | 663280 | 666598 | C | B1 | 886940 | 887111 |  |
| D10 | 899936 | 900542 | M | F1 | 777033 | 780930 | C | B1 | 1013189 | 1016971 | M |
| D10 | 919005 | 919006 |  | F1 | 782251 | 782252 | C | B1 | 1814949 | 1817116 | M |
| D10 | 919753 | 919921 |  | F1 | 789243 | 792073 | C | B1 | 2115413 | 2122425 |  |
| D10 | 925359 | 927275 | C | F1 | 1372913 | 1372950 | M | B2 | 309799 | 320920 | M |
| D10 | 928567 | 929889 | C | F1 | 1969550 | 1969551 |  | B2 | 663280 | 663601 | C |
| D10 | 1001158 | 1001167 |  | F1 | 2110426 | 2120427 | C | B2 | 674577 | 674647 | C |
| D10 | 1001821 | 1001822 |  | F1 | 2121161 | 2122425 | C | B2 | 681901 | 682265 |  |
| D11 | 774167 | 777725 |  | F2 | 95 | 5813 | C | B2 | 699788 | 701685 |  |
| D11 | 804167 | 805044 | M | F2 | 6068 | 14149 | C | B2 | 703856 | 703857 |  |
| D11 | 811510 | 812179 |  | F2 | 561730 | 561734 |  | B2 | 767614 | 771549 | C |
| D11 | 2110426 | 2118576 |  | F2 | 566621 | 569914 |  | B2 | 773305 | 777940 | C |
| D12 | 4205 | 4961 | C | F2 | 764551 | 780623 |  | B2 | 791138 | 799800 | C |
| D12 | 8990 | 9008 | C | F2 | 789243 | 791264 | M | B2 | 1370180 | 1374080 | M |
| D12 | 75634 | 76271 | M | F2 | 893127 | 893139 | M | B2 | 1465687 | 1477478 | C |
| D12 | 230920 | 231010 | C | F2 | 896815 | 896816 | C | B2 | 1484135 | 1487702 | C |
| D12 | 235017 | 237792 | C | F2 | 901319 | 901320 | C | B2 | 1693969 | 1698617 | C |
| D12 | 409752 | 411838 | C | F2 | 987550 | 987551 |  | B2 | 1698778 | 1699973 | C |
| D12 | 413141 | 414165 | C | F2 | 1170127 | 1171627 | M | B2 | 1788933 | 1788934 |  |
| D12 | 499234 | 499347 |  | F2 | 1182993 | 1185935 | C | B2 | 1915391 | 1915392 |  |
| D12 | 777033 | 777940 |  | F2 | 1187261 | 1201922 | C | B4 | 281104 | 282439 | M |
| D12 | 1176628 | 1181043 | M | F2 | 1209306 | 1209372 |  | B4 | 309535 | 311783 |  |
| D12 | 1424914 | 1427836 | M | F2 | 1298334 | 1298335 |  | B4 | 502960 | 503368 | C |
| D12 | 2046679 | 2046680 | C | F2 | 1752468 | 1753118 | C | B4 | 508233 | 508526 | C |
| D12 | 2047821 | 2047827 | C | F2 | 1753527 | 1753572 | C | B4 | 715711 | 717030 |  |
| D12 | 2115413 | 2118576 |  | F2 | 1753681 | 1753690 | C | B4 | 777033 | 777940 | C |
| D14 | 10903 | 11154 |  | F2 | 2103350 | 2122425 | M | B4 | 780930 | 781047 | C |
| D14 | 91726 | 91727 |  | F4 | 95 | 96 |  | B4 | 782212 | 782251 | C |
| D14 | 101429 | 102954 | M | F4 | 878 | 1430 | C | B4 | 782838 | 785328 | C |
| D14 | 137651 | 140115 | M | F4 | 1618 | 1646 | C | B4 | 786691 | 787819 | C |
| D14 | 345259 | 346806 | M | F4 | 777033 | 780930 | C | B4 | 1979782 | 1982987 |  |
| D14 | 769140 | 769217 | C | F4 | 782251 | 782252 | C | B4 | 2118576 | 2122425 |  |
| D14 | 777033 | 780623 | C | F4 | 782838 | 786047 | C | B6 | 8143 | 22050 |  |
| D14 | 1066784 | 1069263 | M | F4 | 786691 | 789689 | C | B6 | 309535 | 309536 |  |
| D14 | 1198884 | 1198885 |  | F4 | 790757 | 809961 | C | B6 | 311783 | 315200 |  |
| D14 | 1770389 | 1771019 | C | F4 | 811510 | 830999 | C | B6 | 375621 | 375635 |  |
| D14 | 1771204 | 1771527 | C | F4 | 837192 | 842118 | C | B6 | 393360 | 393361 |  |
| D14 | 1786148 | 1787158 | C | F4 | 846555 | 858745 | C | B6 | 693382 | 701685 |  |
| D14 | 1966148 | 1966637 |  | F4 | 863820 | 868474 | C | B6 | 734647 | 739858 |  |
| D14 | 2016041 | 2016293 | C | F4 | 868571 | 872179 | C | B6 | 767614 | 781047 |  |
| D14 | 2016425 | 2016925 | C | F4 | 920756 | 923502 | M | B6 | 781775 | 782251 |  |
| D14 | 2017181 | 2017784 | C | F4 | 1976645 | 1981082 | C | B6 | 782387 | 786047 |  |
| D15 | 102819 | 103335 |  | F4 | 1981324 | 1986682 | C | B6 | 786691 | 794175 |  |
| D15 | 122643 | 122644 |  | F4 | 2063214 | 2064071 | C | B6 | 794540 | 803803 |  |
| D15 | 771207 | 777940 |  | F4 | 2064098 | 2065483 | C | B6 | 1008115 | 1008116 |  |
| D15 | 1156329 | 1156330 |  | F4 | 2083786 | 2110426 |  | B6 | 1009773 | 1010238 |  |
| D15 | 1208470 | 1209372 |  | F4 | 2115413 | 2122425 |  | B6 | 1011230 | 1012106 |  |
| D15 | 2112154 | 2122425 |  | F5 | 303331 | 303332 |  | B6 | 1012841 | 1014322 |  |
| D17 | 116082 | 117862 | M | F5 | 375621 | 375978 | C | B6 | 1019396 | 1023833 |  |
| D17 | 330249 | 331242 | M | F5 | 376329 | 378428 | C | B6 | 1210352 | 1212174 |  |
| D17 | 557903 | 557984 |  | F5 | 378542 | 380875 | C | B6 | 1280785 | 1284891 |  |
| D17 | 661260 | 661335 | C | F5 | 380898 | 381012 | C | B6 | 1536561 | 1549032 |  |
| D17 | 661502 | 663466 | C | F5 | 399371 | 412082 | M | B6 | 2060375 | 2063100 |  |
| D17 | 663601 | 663817 | C | F5 | 772069 | 780930 | M | B8 | 4205 | 8250 | M |
| D17 | 772467 | 772512 | C | F5 | 812749 | 817906 |  | B8 | 690725 | 690726 | C |
| D17 | 777033 | 777940 | C | F5 | 818238 | 819699 |  | B8 | 691236 | 691860 | C |
| D17 | 986502 | 988290 | M | F5 | 827645 | 827648 |  | B8 | 692881 | 701685 | C |
| D17 | 1549614 | 1550321 | M | F5 | 841893 | 842118 |  | B8 | 826592 | 827321 |  |
| D17 | 1925981 | 1930403 | M | F5 | 1248073 | 1257477 |  | B8 | 1141168 | 1141172 |  |
| D17 | 2113538 | 2121710 |  | F5 | 1257887 | 1257888 | C | B8 | 1143020 | 1143223 |  |
| D18 | 313971 | 314313 |  | F5 | 1257932 | 1271905 | C | B8 | 1917433 | 1921488 | M |
| D18 | 777033 | 779917 |  | F5 | 1273217 | 1273268 |  | B8 | 2025173 | 2025475 |  |
| D18 | 1175755 | 1175756 |  | F5 | 1284891 | 1289882 |  | B8 | 2046324 | 2046679 | M |
| D18 | 2115413 | 2118576 |  | F5 | 1383080 | 1398322 | M | B8 | 2077798 | 2083527 | C |
| D19 | 777033 | 779917 |  | F5 | 1900054 | 1903417 | M | B8 | 2083719 | 2084550 | C |
| D19 | 838147 | 842118 | M | F5 | 2114786 | 2122209 |  | B8 | 2103662 | 2103720 |  |
| D19 | 1416568 | 1418731 | M | F6 | 95 | 3334 | C | B8 | 2115413 | 2121710 |  |
| D19 | 1801607 | 1807733 |  | F6 | 10489 | 23674 | C | B9 | 95 | 4961 | M |
| D19 | 1838048 | 1838061 |  | F6 | 258724 | 263337 | M | B9 | 108791 | 111816 | M |
| D19 | 2021680 | 2021681 |  | F6 | 777033 | 780930 | C | B9 | 692158 | 701685 | M |
| D19 | 2115413 | 2118576 |  | F6 | 782251 | 782252 | C | B9 | 736493 | 750612 | C |
| D20 | 95 | 659 |  | F6 | 782838 | 786047 | C | B9 | 750896 | 763892 | C |
| D20 | 777033 | 777150 |  | F6 | 786691 | 789243 | C | B9 | 777033 | 781047 | C |
| D20 | 1862119 | 1863939 | M | F6 | 811510 | 816451 |  | B9 | 781775 | 782251 | C |
| D20 | 2107330 | 2122425 |  | F6 | 2099449 | 2111687 |  | B9 | 782838 | 786047 | C |
| D21 | 659 | 2757 | M | F6 | 2112154 | 2122425 |  | B9 | 786691 | 789243 | C |
| D21 | 293828 | 294503 |  | F7 | 770952 | 777940 | C | B9 | 794175 | 805711 | C |
| D21 | 415846 | 415847 |  | F7 | 787753 | 791682 | C | B9 | 811510 | 820256 | C |
| D21 | 537062 | 537122 |  | F7 | 1695668 | 1695669 |  | B9 | 1728054 | 1732965 | M |
| D21 | 610227 | 610369 | M | F7 | 1703959 | 1707658 | M | B9 | 1944690 | 1950550 | M |
| D21 | 774167 | 777150 |  | F7 | 2118576 | 2120427 |  | B9 | 2031604 | 2035909 | M |
| D21 | 1461903 | 1465283 | M | F7 | 2121161 | 2121162 |  | B9 | 2110426 | 2122425 |  |
| D21 | 1575679 | 1576245 | M | F8 | 95 | 878 |  | B10 | 760281 | 762925 |  |
| D21 | 1707457 | 1708828 |  | F8 | 765967 | 780930 |  | B10 | 764290 | 765031 |  |
| D21 | 1965322 | 1967526 | M | F8 | 954626 | 958485 | C | B10 | 777033 | 777725 |  |
| D21 | 2028230 | 2028738 | C | F8 | 958583 | 961634 | C | B10 | 784719 | 786047 | M |
| D21 | 2031785 | 2037464 | C | F8 | 1239895 | 1243157 | C | B10 | 1122983 | 1123343 |  |
| D21 | 2118576 | 2121161 |  | F8 | 1244017 | 1250947 | C | B11 | 79718 | 87408 | M |
| D23 | 95 | 4205 |  | F8 | 2104406 | 2120427 |  | B11 | 292769 | 295047 |  |
| D23 | 213317 | 213318 |  | F8 | 2121161 | 2122425 |  | B11 | 456745 | 460490 | M |
| D23 | 333854 | 335517 | M | F9 | 269339 | 269340 |  | B11 | 770081 | 781047 |  |
| D23 | 769589 | 777940 |  | F9 | 777033 | 777725 |  | B11 | 782212 | 782251 |  |
| D23 | 1288520 | 1289654 |  | F9 | 1946795 | 1948253 | M | B11 | 782838 | 786047 |  |
| D23 | 1290266 | 1290267 |  | F9 | 1970817 | 1972210 | M | B11 | 787228 | 787229 |  |
| D23 | 2115413 | 2120427 | C | F9 | 2107499 | 2112411 | M | B11 | 1678319 | 1679859 |  |
| D23 | 2121161 | 2122425 | C | F9 | 2118576 | 2121161 |  | B11 | 1962536 | 1962710 |  |
| D24 | 777033 | 777150 |  | F10 | 95 | 4205 |  | B11 | 1971184 | 1972210 |  |
| D24 | 2115413 | 2118576 |  | F10 | 636374 | 636381 |  | B11 | 2043238 | 2050206 |  |
| D25 | 777033 | 777150 | C | F10 | 758408 | 764015 | M | B11 | 2060375 | 2063235 | M |
| D25 | 779764 | 780930 | C | F10 | 771207 | 777940 | C | B12 | 3871 | 4918 | M |
| D25 | 782251 | 782252 | C | F10 | 779917 | 780930 | C | B12 | 771368 | 777940 |  |
| D25 | 785051 | 785052 | C | F10 | 2111663 | 2122425 |  | B12 | 851919 | 851992 |  |
| D25 | 786047 | 786048 | C | F11 | 242043 | 245173 | C | B12 | 1505956 | 1510073 | M |
| D25 | 786691 | 791682 | C | F11 | 245708 | 249631 | C | B13 | 1048564 | 1051173 |  |
| D25 | 799800 | 800074 | C | F11 | 250232 | 251109 | C | B13 | 2033100 | 2033101 |  |
| D25 | 809749 | 809961 |  | F11 | 771934 | 777150 |  | B14 | 696402 | 701685 |  |
| D25 | 811510 | 812002 |  | F11 | 1854545 | 1854672 | M | B14 | 1185722 | 1191528 | C |
| D25 | 922080 | 929239 | M | F11 | 1858759 | 1858760 |  | B14 | 1191742 | 1221023 | C |
| D25 | 1311955 | 1313015 | M | F11 | 2076513 | 2082771 | M | B14 | 1261116 | 1261384 |  |
| D25 | 1432025 | 1433796 | M | F11 | 2093664 | 2094180 | C | B14 | 1279882 | 1279883 |  |
| D25 | 2118576 | 2120427 | C | F11 | 2094959 | 2108389 | C | B14 | 1299783 | 1302059 | M |
| D25 | 2121161 | 2122425 | C | F11 | 2113815 | 2118576 | C | B14 | 1364474 | 1367524 | M |
| D26 | 289923 | 290007 | C | F12 | 95 | 2757 |  | B14 | 1404297 | 1421630 |  |
| D26 | 292190 | 292796 | C | F12 | 770952 | 780623 |  | B14 | 1449138 | 1452278 | C |
| D26 | 292844 | 293146 | C | F12 | 1232970 | 1244164 | M | B14 | 1452367 | 1455854 | C |
| D26 | 350852 | 351718 | M | F12 | 1262413 | 1268193 |  | B14 | 1512182 | 1514819 | M |
| D26 | 493927 | 493928 |  | F12 | 1269762 | 1270380 |  | B14 | 1519514 | 1533355 | M |
| D26 | 777033 | 777940 |  | F12 | 1372913 | 1372989 | M | B14 | 1604037 | 1608990 | M |
| D26 | 1711123 | 1715051 | C | F12 | 1679521 | 1679522 |  | B14 | 1929893 | 1938295 | M |
| D26 | 1715249 | 1716422 | C | F12 | 2112411 | 2122425 |  | B14 | 2107330 | 2121710 |  |
| D26 | 1986639 | 1988338 | C | F13 | 95 | 2089 |  | B15 | 771708 | 777940 |  |
| D26 | 1991882 | 1994705 | C | F13 | 9282 | 11964 | M | B15 | 1306659 | 1306660 |  |
| D26 | 2118576 | 2118577 |  | F13 | 62293 | 62728 | C | B15 | 1306696 | 1306697 |  |
| D27 | 777033 | 780623 |  | F13 | 64066 | 67922 | C | B15 | 2021680 | 2023883 | C |
| D27 | 2118576 | 2118577 |  | F13 | 237179 | 240877 | C | B15 | 2025475 | 2029506 | C |
| D28 | 165811 | 167507 | C | F13 | 241163 | 245708 | C | B16 | 691115 | 701685 |  |
| D28 | 168618 | 170430 | C | F13 | 645137 | 648960 |  | B16 | 703856 | 703857 |  |
| D28 | 760759 | 762553 | C | F13 | 668686 | 670156 | M | B16 | 1031124 | 1036919 | M |
| D28 | 762594 | 763013 | C | F13 | 770952 | 780623 |  | B16 | 1062646 | 1070096 | M |
| D28 | 767614 | 768408 | C | F13 | 1121701 | 1124990 |  | B16 | 1188142 | 1188143 |  |
| D28 | 777033 | 780930 |  | F13 | 1355926 | 1355927 |  | B16 | 1189403 | 1189404 |  |
| D28 | 1763949 | 1767243 | M | F13 | 1453230 | 1455253 | M | B16 | 1296867 | 1306059 | M |
| D28 | 1883936 | 1885178 | M | F13 | 1464823 | 1467439 | C | B16 | 1844779 | 1845556 | M |
| D28 | 2115413 | 2121161 |  | F13 | 1472858 | 1476527 | C | B17 | 693266 | 701206 | M |
| D29 | 659 | 2757 | M | F13 | 1763949 | 1766273 | M | B17 | 721523 | 724061 | M |
| D29 | 293828 | 294503 |  | F13 | 2115413 | 2122425 |  | B17 | 760759 | 777940 |  |
| D29 | 415846 | 415847 |  | F14 | 95 | 3334 |  | B17 | 1024221 | 1030357 | M |
| D29 | 537062 | 537122 |  | F14 | 7225 | 11964 |  | B17 | 1261116 | 1261384 |  |
| D29 | 610227 | 610369 | M | F14 | 109849 | 110287 |  | B17 | 1805232 | 1805233 |  |
| D29 | 774167 | 777150 |  | F14 | 772477 | 777940 |  | B18 | 747838 | 754824 | M |
| D29 | 1461903 | 1465283 | M | F14 | 1938294 | 1938295 |  | B18 | 1035542 | 1041989 | M |
| D29 | 1575679 | 1576245 | M | F14 | 1982987 | 1986682 | M | B18 | 1316042 | 1317429 | C |
| D29 | 1707457 | 1707991 | M | F14 | 2110426 | 2122425 |  | B18 | 1318597 | 1327008 | C |
| D29 | 2028230 | 2028738 | C | F15 | 93942 | 94283 |  | B18 | 1358352 | 1359059 | M |
| D29 | 2031785 | 2037464 | C | F15 | 131910 | 131970 |  | B18 | 1411192 | 1412323 | M |
| D29 | 2118576 | 2121161 |  | F15 | 764251 | 765979 | M | B18 | 1734278 | 1735968 |  |
| D30 | 277433 | 278164 |  | F15 | 772994 | 777940 | C | B18 | 1737272 | 1737638 |  |
| D30 | 777033 | 777940 |  | F15 | 790604 | 805511 | C | B18 | 1826006 | 1827523 | M |
| D30 | 814322 | 814323 |  | F15 | 807475 | 809961 | C | B18 | 1966148 | 1968081 | M |
| D30 | 1581339 | 1584602 | M | F15 | 2111663 | 2118576 |  | B19 | 752016 | 753422 | M |
| D30 | 1843084 | 1843086 | C | F16 | 95 | 1646 |  | B19 | 759909 | 759910 |  |
| D30 | 1845190 | 1845279 | C | F16 | 5514 | 7292 | C | B19 | 768965 | 770277 | C |
| D30 | 2112154 | 2120427 |  | F16 | 8143 | 14149 | C | B19 | 771483 | 777940 | C |
| D31 | 5514 | 6326 | M | F16 | 585631 | 589558 | M | B19 | 1039993 | 1052589 | C |
| D31 | 271133 | 272447 |  | F16 | 777033 | 780930 | C | B19 | 1053417 | 1056828 | C |
| D31 | 772994 | 777150 |  | F16 | 784719 | 785328 | C | B19 | 1072291 | 1072481 |  |
| D31 | 1380074 | 1380603 |  | F16 | 786691 | 797558 | C | B19 | 1525902 | 1526696 | M |
| D31 | 1392429 | 1393462 | M | F16 | 1243983 | 1248073 | M | B19 | 2056323 | 2060862 | M |
| D31 | 1693205 | 1698080 | C | F16 | 1351118 | 1356046 | M | B20 | 667972 | 672078 | M |
| D31 | 1698098 | 1699973 | C | F16 | 1678606 | 1678607 |  | B20 | 672763 | 672764 |  |
| D31 | 1704570 | 1704648 | C | F16 | 1679521 | 1679859 |  | B20 | 683695 | 685124 |  |
| D31 | 1705822 | 1707658 |  | F16 | 1969550 | 1970291 | C | B20 | 685824 | 693382 |  |
| D31 | 1813530 | 1816403 | M | F16 | 1971391 | 1972210 | C | B20 | 693656 | 693791 | C |
| D31 | 1886465 | 1886466 |  | F16 | 1974074 | 1975088 | C | B20 | 693825 | 701685 | C |
| D31 | 1912862 | 1912875 |  | F16 | 1975605 | 1975814 | C | B20 | 703856 | 703857 |  |
| D31 | 2114786 | 2118576 |  | F16 | 2115413 | 2118576 | C | B20 | 764290 | 777150 |  |
| D32 | 289923 | 290007 | C | F16 | 2121487 | 2122336 | C | B20 | 793213 | 802434 |  |
| D32 | 292190 | 292796 | C | F17 | 95 | 96 |  | B20 | 857236 | 864796 | M |
| D32 | 292844 | 293146 | C | F17 | 878 | 1430 | C | B20 | 1343022 | 1343023 |  |
| D32 | 350852 | 351718 | M | F17 | 1618 | 4721 | C | B20 | 1387451 | 1398848 | M |
| D32 | 493927 | 493928 |  | F17 | 67583 | 67584 |  | B20 | 1698509 | 1705500 |  |
| D32 | 777033 | 777940 |  | F17 | 71121 | 71127 | C | B20 | 1707897 | 1710113 |  |
| D32 | 1711123 | 1715051 | C | F17 | 71360 | 71361 | C | B20 | 1788933 | 1788938 |  |
| D32 | 1715249 | 1716422 | C | F17 | 165528 | 167555 | M | B20 | 1801607 | 1804086 |  |
| D32 | 1986639 | 1988338 | C | F17 | 290240 | 295431 |  | B20 | 1804375 | 1807445 |  |
| D32 | 1991882 | 1992469 | C | F17 | 771368 | 777940 |  | B21 | 772069 | 772340 | C |
| D32 | 1992544 | 1994705 | C | F17 | 944174 | 945509 | M | B21 | 772512 | 772994 | C |
| D32 | 2118576 | 2118577 |  | F17 | 1225006 | 1227323 | C | B21 | 777033 | 777150 | C |
| D38 | 777033 | 777940 |  | F17 | 1227482 | 1229282 | C | B21 | 1788933 | 1788934 |  |
| D38 | 2118576 | 2118577 |  | F17 | 1902418 | 1903772 | M | B22 | 534851 | 534858 | C |
|  |  |  |  | F17 | 2118576 | 2120427 | C | B22 | 534957 | 537122 | C |
|  |  |  |  | F17 | 2121161 | 2122425 | C | B22 | 777033 | 779917 |  |
|  |  |  |  | F18 | 95 | 3499 | M | B22 | 1128002 | 1131161 | M |
|  |  |  |  | F18 | 164734 | 169251 | C | B22 | 1163212 | 1166257 |  |
|  |  |  |  | F18 | 169762 | 170430 | C | B22 | 1252961 | 1257622 | C |
|  |  |  |  | F18 | 205781 | 207373 |  | B22 | 1257932 | 1258090 | C |
|  |  |  |  | F18 | 777033 | 780930 | C | B22 | 1258252 | 1260103 | C |
|  |  |  |  | F18 | 782251 | 782252 | C | B22 | 1336835 | 1337773 | C |
|  |  |  |  | F18 | 782838 | 785051 | C | B22 | 1339588 | 1341372 | C |
|  |  |  |  | F18 | 786047 | 786048 | C | B23 | 693266 | 693267 |  |
|  |  |  |  | F18 | 804167 | 806772 |  | B23 | 777033 | 781047 |  |
|  |  |  |  | F18 | 820256 | 833072 | M | B23 | 833449 | 844656 |  |
|  |  |  |  | F18 | 1121338 | 1122983 |  | B23 | 844784 | 844785 |  |
|  |  |  |  | F18 | 1527238 | 1528554 | M | B23 | 962699 | 964706 |  |
|  |  |  |  | F18 | 1987579 | 1988507 | M | B23 | 1029275 | 1029962 | M |
|  |  |  |  | F18 | 2118576 | 2122425 |  | B23 | 1050279 | 1055175 |  |
|  |  |  |  | F19 | 2089 | 2757 | C | B23 | 1228524 | 1242467 | M |
|  |  |  |  | F19 | 3499 | 4918 | C | B23 | 1356514 | 1356515 |  |
|  |  |  |  | F19 | 8143 | 11964 | C | B23 | 1523588 | 1529119 |  |
|  |  |  |  | F19 | 773104 | 780930 | C | B23 | 1693334 | 1694599 | M |
|  |  |  |  | F19 | 782251 | 782252 | C | B24 | 771368 | 777940 |  |
|  |  |  |  | F19 | 2113538 | 2118576 |  | B24 | 986850 | 989420 | M |
|  |  |  |  | F20 | 95 | 878 |  | B24 | 1034137 | 1034927 | M |
|  |  |  |  | F20 | 756386 | 761664 | C | B25 | 510797 | 512369 | C |
|  |  |  |  | F20 | 767614 | 780930 | C | B25 | 512568 | 521519 | C |
|  |  |  |  | F20 | 782251 | 782252 | C | B25 | 521898 | 522794 | C |
|  |  |  |  | F20 | 782838 | 786047 | C | B25 | 600565 | 602701 | M |
|  |  |  |  | F20 | 786691 | 795630 | C | B25 | 745664 | 747838 | C |
|  |  |  |  | F20 | 2093664 | 2102078 | C | B25 | 747969 | 777940 | C |
|  |  |  |  | F20 | 2103350 | 2105540 | C | B25 | 1341564 | 1342560 | C |
|  |  |  |  | F20 | 2115413 | 2122425 |  | B25 | 1342666 | 1343343 | C |
|  |  |  |  | F21 | 95 | 1430 |  | B25 | 1343732 | 1346674 | C |
|  |  |  |  | F21 | 251966 | 272447 |  | B25 | 1346907 | 1346908 | C |
|  |  |  |  | F21 | 765031 | 779764 |  | B25 | 1347991 | 1353187 | C |
|  |  |  |  | F21 | 830814 | 830815 |  | B25 | 1411255 | 1411256 |  |
|  |  |  |  | F21 | 1586857 | 1591038 |  | B25 | 1437393 | 1447800 | M |
|  |  |  |  | F21 | 2115413 | 2121710 | C | B25 | 1471842 | 1471843 |  |
|  |  |  |  | F21 | 2122329 | 2122425 | C | B25 | 1799716 | 1800302 | M |
|  |  |  |  | F23 | 95 | 96 |  |  |  |  |  |
|  |  |  |  | F23 | 878 | 4918 |  |  |  |  |  |
|  |  |  |  | F23 | 5514 | 14149 |  |  |  |  |  |
|  |  |  |  | F23 | 764479 | 780930 |  |  |  |  |  |
|  |  |  |  | F23 | 782251 | 782252 |  |  |  |  |  |
|  |  |  |  | F23 | 797081 | 801891 | M |  |  |  |  |
|  |  |  |  | F23 | 1312715 | 1312787 | C |  |  |  |  |
|  |  |  |  | F23 | 1312916 | 1312922 | C |  |  |  |  |
|  |  |  |  | F23 | 1313015 | 1317266 | C |  |  |  |  |
|  |  |  |  | F23 | 1322782 | 1324493 | C |  |  |  |  |
|  |  |  |  | F23 | 1329324 | 1330153 | C |  |  |  |  |
|  |  |  |  | F23 | 1343460 | 1346674 | M |  |  |  |  |
|  |  |  |  | F23 | 2012026 | 2013778 | M |  |  |  |  |
|  |  |  |  | F23 | 2021680 | 2022400 | C |  |  |  |  |
|  |  |  |  | F23 | 2022511 | 2022512 | C |  |  |  |  |
|  |  |  |  | F23 | 2023312 | 2033141 | C |  |  |  |  |
|  |  |  |  | F23 | 2038202 | 2039154 | C |  |  |  |  |
|  |  |  |  | F23 | 2115413 | 2118576 | C |  |  |  |  |
|  |  |  |  | F23 | 2122329 | 2122425 | C |  |  |  |  |
|  |  |  |  | F24 | 95 | 3871 |  |  |  |  |  |
|  |  |  |  | F24 | 304069 | 316015 | M |  |  |  |  |
|  |  |  |  | F24 | 580946 | 593852 | M |  |  |  |  |
|  |  |  |  | F24 | 768965 | 780930 | C |  |  |  |  |
|  |  |  |  | F24 | 782251 | 782252 | C |  |  |  |  |
|  |  |  |  | F24 | 791682 | 797558 | C |  |  |  |  |
|  |  |  |  | F24 | 1938294 | 1938295 |  |  |  |  |  |
|  |  |  |  | F24 | 2085376 | 2088006 | M |  |  |  |  |
|  |  |  |  | F24 | 2101512 | 2102078 | C |  |  |  |  |
|  |  |  |  | F24 | 2103350 | 2105540 | C |  |  |  |  |
|  |  |  |  | F24 | 2107499 | 2110426 | C |  |  |  |  |
|  |  |  |  | F24 | 2115413 | 2122425 | C |  |  |  |  |
