# Supplementary material for: Evolution via recombination: Cell-to-cell contact facilitates larger recombination events in Streptococcus pneumoniae
Source: PLoS Genet. 2018 Jun 13;14(6):e1007410. doi: 10.1371/journal.pgen.1007410 (PMC6016952; doi:10.1371/journal.pgen.1007410)
Supplement: S6 Table — (DOCX) [file pgen.1007410.s008.docx]

**Table S6. Assembled statistics of the recombination events identified in each recombinant**.

| **Class** | **Recombinant strain** | **Number of recombination events** | **Mean size** | **Standard Deviation** | **Number of events > 10,000 bp** | **Total Transferred (a)** | **Donor/total SNPs Transferred (b)** |
| --- | --- | --- | --- | --- | --- | --- | --- |
| **Saturating DNA** | **D3** | 4 | 6661 | 6027 | 1 | 1.26% | 0.52% |
|  | **D10** | 10 | 827 | 1336 | 0 | 0.39% | 0.50% |
|  | **D11** | 4 | 3314 | 3482 | 0 | 0.62% | 0.30% |
|  | **D12** | 14 | 1351 | 1452 | 0 | 0.89% | 0.73% |
|  | **D14** | 16 | 984 | 1060 | 0 | 0.74% | 0.81% |
|  | **D15** | 6 | 3070 | 4367 | 1 | 0.87% | 0.47% |
|  | **D17** | 12 | 1763 | 2367 | 0 | 1.00% | 1.13% |
|  | **D18** | 4 | 1597 | 1657 | 0 | 0.30% | 0.09% |
|  | **D19** | 7 | 2617 | 2174 | 0 | 0.86% | 0.51% |
|  | **D20** | 4 | 4399 | 7167 | 1 | 0.83% | 0.30% |
|  | **D21** | 13 | 1712 | 1668 | 0 | 1.05% | 0.78% |
|  | **D23** | 8 | 2692 | 2914 | 0 | 1.01% | 0.50% |
|  | **D24** | 2 | 1640 | 2154 | 0 | 0.15% | 0.04% |
|  | **D25** | 14 | 1454 | 2105 | 0 | 0.96% | 1.13% |
|  | **D26** | 11 | 1126 | 1255 | 0 | 0.58% | 1.39% |
|  | **D27** | 2 | 1795 | 2539 | 0 | 0.17% | 0.06% |
|  | **D28** | 9 | 2300 | 1703 | 0 | 0.97% | 0.44% |
|  | **D29** | 12 | 1601 | 1765 | 0 | 0.90% | 0.68% |
|  | **D30** | 7 | 1895 | 3036 | 0 | 0.62% | 0.70% |
|  | **D31** | 13 | 1783 | 1657 | 0 | 1.09% | 1.28% |
|  | **D32** | 12 | 1026 | 1133 | 0 | 0.58% | 1.39% |
|  | **D38** | 2 | 454 | 641 | 0 | 0.04% | 0.04% |
| **Biofilm** | **B1** | 10 | 4620 | 3636 | 1 | 2.18% | 2.25% |
|  | **B2** | 16 | 3507 | 3939 | 2 | 2.64% | 3.17% |
|  | **B4** | 12 | 1445 | 1250 | 0 | 0.82% | 0.80% |
|  | **B6** | 21 | 4455 | 4617 | 3 | 4.41% | 3.31% |
|  | **B8** | 14 | 2288 | 2927 | 0 | 1.51% | 1.66% |
|  | **B9** | 15 | 6809 | 4309 | 4 | 4.81% | 4.05% |
|  | **B10** | 5 | 1153 | 903 | 0 | 0.27% | 0.34% |
|  | **B11** | 12 | 3375 | 3470 | 1 | 1.91% | 1.94% |
|  | **B12** | 4 | 2952 | 2965 | 0 | 0.56% | 0.36% |
|  | **B13** | 2 | 1305 | 1845 | 0 | 0.12% | 0.13% |
|  | **B14** | 15 | 7609 | 7964 | 4 | 5.38% | 6.39% |
|  | **B15** | 5 | 2493 | 2686 | 0 | 0.59% | 0.30% |
|  | **B16** | 8 | 4223 | 4523 | 1 | 1.59% | 2.37% |
|  | **B17** | 6 | 5677 | 6465 | 1 | 1.60% | 1.57% |
|  | **B18** | 10 | 3075 | 2988 | 0 | 1.44% | 1.44% |
|  | **B19** | 9 | 3412 | 4065 | 1 | 1.45% | 1.34% |
|  | **B20** | 17 | 4523 | 4308 | 2 | 3.62% | 3.31% |
|  | **B21** | 4 | 218 | 208 | 0 | 0.04% | 0.05% |
|  | **B22** | 10 | 2065 | 1447 | 0 | 0.97% | 1.62% |
|  | **B23** | 11 | 3959 | 4737 | 2 | 2.05% | 2.21% |
|  | **B24** | 3 | 3311 | 2961 | 0 | 0.47% | 0.24% |
|  | **B25** | 15 | 4434 | 7754 | 2 | 3.13% | 3.37% |
| **Filter Assemblage** | **F1** | 14 | 3044 | 4163 | 2 | 2.01% | 1.84% |
|  | **F2** | 19 | 3903 | 6110 | 3 | 3.49% | 3.22% |
|  | **F4** | 20 | 6160 | 7432 | 4 | 5.80% | 3.67% |
|  | **F5** | 19 | 4619 | 5168 | 3 | 4.13% | 3.33% |
|  | **F6** | 10 | 5815 | 4462 | 3 | 2.74% | 1.40% |
|  | **F7** | 6 | 2745 | 2690 | 0 | 0.78% | 0.38% |
|  | **F8** | 8 | 6267 | 5994 | 2 | 2.36% | 1.61% |
|  | **F9** | 6 | 1840 | 1734 | 0 | 0.52% | 0.24% |
|  | **F10** | 6 | 4705 | 3941 | 1 | 1.33% | 0.98% |
|  | **F11** | 10 | 3824 | 4075 | 1 | 1.80% | 1.00% |
|  | **F12** | 8 | 5002 | 4779 | 2 | 1.89% | 1.20% |
|  | **F13** | 16 | 3319 | 2372 | 0 | 2.50% | 2.36% |
|  | **F14** | 7 | 4225 | 3993 | 1 | 1.39% | 0.78% |
|  | **F15** | 7 | 4483 | 5213 | 1 | 1.48% | 0.91% |
|  | **F16** | 17 | 2634 | 2816 | 1 | 2.11% | 1.35% |
|  | **F17** | 15 | 1825 | 1918 | 0 | 1.29% | 1.40% |
|  | **F18** | 14 | 2818 | 3223 | 1 | 1.86% | 1.20% |
|  | **F19** | 6 | 3129 | 2999 | 0 | 0.88% | 0.38% |
|  | **F20** | 9 | 5460 | 4365 | 1 | 2.31% | 1.15% |
|  | **F21** | 7 | 6732 | 7940 | 2 | 2.22% | 1.97% |
|  | **F23** | 19 | 3186 | 4314 | 1 | 2.85% | 1.81% |
|  | **F24** | 12 | 5150 | 4794 | 3 | 2.91% | 1.89% |

**a**. Total bp transferred in all recombination events, as a proportion of the whole genome length.

**b**. Number of SNPs shared between recombinant and donor as a proportion of all donor and recipient genome differentiating SNPs.
